# Redox-active vitamin C suppresses human osteosarcoma growth by triggering intracellular ROS-iron–calcium signaling crosstalk and mitochondrial dysfunction

**DOI:** 10.1016/j.redox.2024.103288

**Published:** 2024-07-26

**Authors:** Prajakta Vaishampayan, Yool Lee

**Affiliations:** a Department of Translational Medicine and Physiology, Elson S. Floyd College of Medicine, Washington State University, Spokane, WA, 99202, USA; b Department of Integrative Physiology and Neuroscience, College of Veterinary Medicine, Washington State University, Pullman, WA, 99164, USA; c Sleep and Performance Research Center, Washington State University, Spokane, WA, 99202, USA; d Steve Gleason Institute for Neuroscience, Washington State University, Spokane, WA, 99202, USA

**Keywords:** Osteosarcoma, Reactive oxygen species, Endoplasmic reticulum oxidoreductase 1 alpha, Inositol 1,4,5-trisphosphate receptors oxidative phosphorylation, Electron transport chain, Mitochondrial dysfunction, ATP

## Abstract

Pharmacological vitamin C (VC) has gained attention for its pro-oxidant characteristics and selective ability to induce cancer cell death. However, defining its role in cancer has been challenging due to its complex redox properties. In this study, using a human osteosarcoma (OS) model, we show that the redox-active property of VC is critical for inducing non-apoptotic cancer cell death via intracellular reactive oxygen species (ROS)-iron-calcium crosstalk and mitochondrial dysfunction. In both 2D and 3D OS cell culture models, only the oxidizable form of VC demonstrated potent dose-dependent cytotoxicity, while non-oxidizable and oxidized VC derivatives had minimal effects. Live-cell imaging showed that only oxidizable VC caused a surge in cytotoxic ROS, dependent on iron rather than copper. Inhibitors of ferroptosis, a form of iron-dependent cell death, along with classical apoptosis inhibitors, were unable to completely counteract the cytotoxic effects induced by VC. Further pharmacological and genetic inhibition analyses showed that VC triggers calcium release through inositol 1,4,5-trisphosphate receptors (IP3Rs), leading to mitochondrial ROS production and eventual cell death. RNA sequencing revealed down-regulation of genes involved in the mitochondrial electron transport chain and oxidative phosphorylation upon pharmacological VC treatment. Consistently, high-dose VC reduced mitochondrial membrane potential, oxidative phosphorylation, and ATP levels, with ATP reconstitution rescuing VC-induced cytotoxicity. In vivo OS xenograft studies demonstrated reduced tumor growth with high-dose VC administration, concomitant with the altered expression of mitochondrial ATP synthase (MT-ATP). These findings emphasize VC's potential clinical utility in osteosarcoma treatment by inducing mitochondrial metabolic dysfunction through a vicious intracellular ROS-iron-calcium cycle.

## 1 Introduction

Osteosarcoma (OS) is the most common primary tumor of the bone and is an especially aggressive, predominantly pediatric cancer that is often fatal in both children and adults [1,2]. Currently, the standard treatment options for OS are surgery, chemotherapy, and radiation therapy, which may be used alone or in combination [3,4]. However, these conventional treatments can result in various adverse effects, such as cardiac toxicity, infertility, and kidney dysfunction [5,6]. Given these limitations, there is increasing demand for new and alternative approaches to treat OS without causing unwanted side effects.

Vitamin C (VC) is a crucial nutrient essential for maintaining cellular physiology [7]. Interestingly, it acts as an antioxidant at physiological concentrations (40–80 μM) in human plasma) and a pro-oxidant at high doses (10–20 mM), suppressing tumor growth [8,9]. The greater sensitivity of tumor cells to high-dose VC has been attributed to their lower capacity to neutralize reactive oxygen species (ROS) [10]. As with other cancer types, increasing studies using osteosarcoma models have shown that high doses of VC can induce cell death independently or synergistically with conventional anti-cancer agents such as cisplatin and arsenic trioxide (ATO) to inhibit tumor cell proliferation and growth [[11], [12], [13], [14], [15], [16]]. Despite the growing evidence supporting the anti-cancer activity of VC, controversy surrounding its role in cancer may arise from its redox properties and dynamic interconversion between its reduced (ascorbic acid, AA) and oxidized (dehydroascorbate, DHA) forms. While current models suggest that DHA is the pharmacologically effective form [17,18], recent studies have shown varied effectiveness of this molecule in cancer cell models [[19], [20], [21]]. In addition, the lack of distinction between AA and DHA in certain previously reported outcomes adds confusion, leaving the contribution of the different redox forms to VC-induced cell death unclear [22,23].

High-doses of VC have been shown to exhibit anti-cancer activity by generating extracellular hydrogen peroxide (H_2_O_2_), impacting redox-dependent signaling and metabolic pathways within cancer cells [19,24]. Catalytic metals, such as copper (Cu^+^/Cu^2+^) or iron (Fe^3+^/Fe^2+^), have been reported to mediate H_2_O_2_ production via ascorbate oxidation [19,24]. Notably, compared to copper, intracellular labile iron has been shown in multiple cancer studies to facilitate increased susceptibility to VC, resulting in preferential cell death relative to normal cells [[25], [26], [27], [28], [29], [30]]. However, the exact involvement of the catalytic metals in VC-induced cytotoxicity in cancer models needs further clarification, as there are increasing numbers of reports with inconsistent findings regarding their roles, depending on the cancer cell type studied [[31], [32], [33], [34]].

In addition to its pro-oxidant properties, VC has been shown to act as a cofactor for Fe^2+^- and 2-oxoglutarate-dependent dioxygenases, including hypoxia-inducible factor (HIF) hydroxylases and DNA demethylases (e.g., ten-eleven translocation enzymes [TET1-3]) [[35], [36], [37]]. While the involvement of these enzymes in tumor suppression is evident in various cancer models when using lower doses of VC (0.1–1 mM) [[38], [39], [40]], it remains unclear if these pathways contribute to acute cancer cell death with higher doses of VC [[39], [40], [41], [42], [43]].

It has been reported that VC's anti-cancer effects involve various cell death mechanisms, including apoptosis, necroptosis, and autophagy, depending on concentration and cell type [44]. Earlier studies suggested the involvement of caspase-dependent apoptosis or necrosis in VC-induced cancer cell death [[45], [46], [47]], but recent evidence indicates the potential involvement of non-canonical mechanisms such as ferroptosis, yielding mixed results [48,49]. In this regard, an increasing number of studies suggest that intracellular Ca^2+^ is a critical mediator of non-apoptotic forms of cell death (necroptosis, ferroptosis, parthanatos, pyroptosis) in response to ROS-inducing compounds [[50], [51], [52]]. However, the precise interaction between intracellular Ca^2+^ and ROS pathways in mediating VC-induced cancer cell death remains to be fully understood.

Pharmacological VC has also been suggested to exert cytotoxic effects in cancer treatment through an additional mechanism involving the disruption of bioenergetics [53,54]. Indeed, recent studies have indicated that the cytotoxicity induced by pharmacological VC is associated with the overactivation of PARP1, triggered by ROS-mediated nuclear DNA damage [55]. This overactivation leads to the consumption of NAD⁺ and subsequent depletion of ATP, ultimately resulting in mitotic cell death [55]. However, subsequent investigations have indicated that the activation of PARP1 in response to ROS-induced DNA damage may not be essential, suggesting the involvement of alternative mechanisms of ATP depletion [55,56]. In addition to cytosolic glycolysis, it has been well-documented that mitochondria play a crucial role in ATP production through oxidative phosphorylation (OXPHOS), which involves the mitochondrial electron transport chain (ETC) complexes (I to IV) whose components are encoded by mitochondrial DNA (mtDNA) [[57], [58], [59]]. However, the precise roles and mechanisms of mitochondrial pathways in VC-induced metabolic alterations and cell death are not yet clearly understood.

In this study, we have shown that redox-reactive VC plays a crucial role in inducing non-apoptotic cell death via intracellular crosstalk between ROS-iron-calcium and mitochondrial metabolic pathways in human OS cells. Using 2D and 3D tumor models, we found that an oxidizable form of VC, relative to its reduced (AA) or oxidized (DHA) derivatives, exerts cancer cell specific cytotoxicity. Furthermore, live-cell analysis combined with pharmacological and genetic perturbation methods revealed that H_2_O_2_ generated by VC upon its oxidation via iron acts in the endoplasmic reticulum (ER) by triggering Ca^2+^ release through IP3Rs and subsequently provoking mitochondrial Ca^2+^ overload and cytotoxic intracellular ROS production. Additional transcriptomic and metabolic analyses revealed that high-dose VC treatment comprehensively down-regulates mitochondrial OXPHOS-regulatory gene expression, which is accompanied by impaired mitochondrial metabolic functions and decreased ATP production. Correspondingly, orthotopic OS xenograft studies revealed that VC treatment reduces tumor growth and results in the marked alteration of mitochondrial ATP synthase expression. Our findings highlight the importance of the intricate interplay between the ROS, iron, and calcium pathways that target mitochondrial functions and metabolism in VC-induced cell death and tumor inhibition in human OS.

## 2 Results

### 2.1 Pharmacological doses of oxidizable vitamin C induce selective cell death in human osteosarcoma cells

**Fig. 1:**
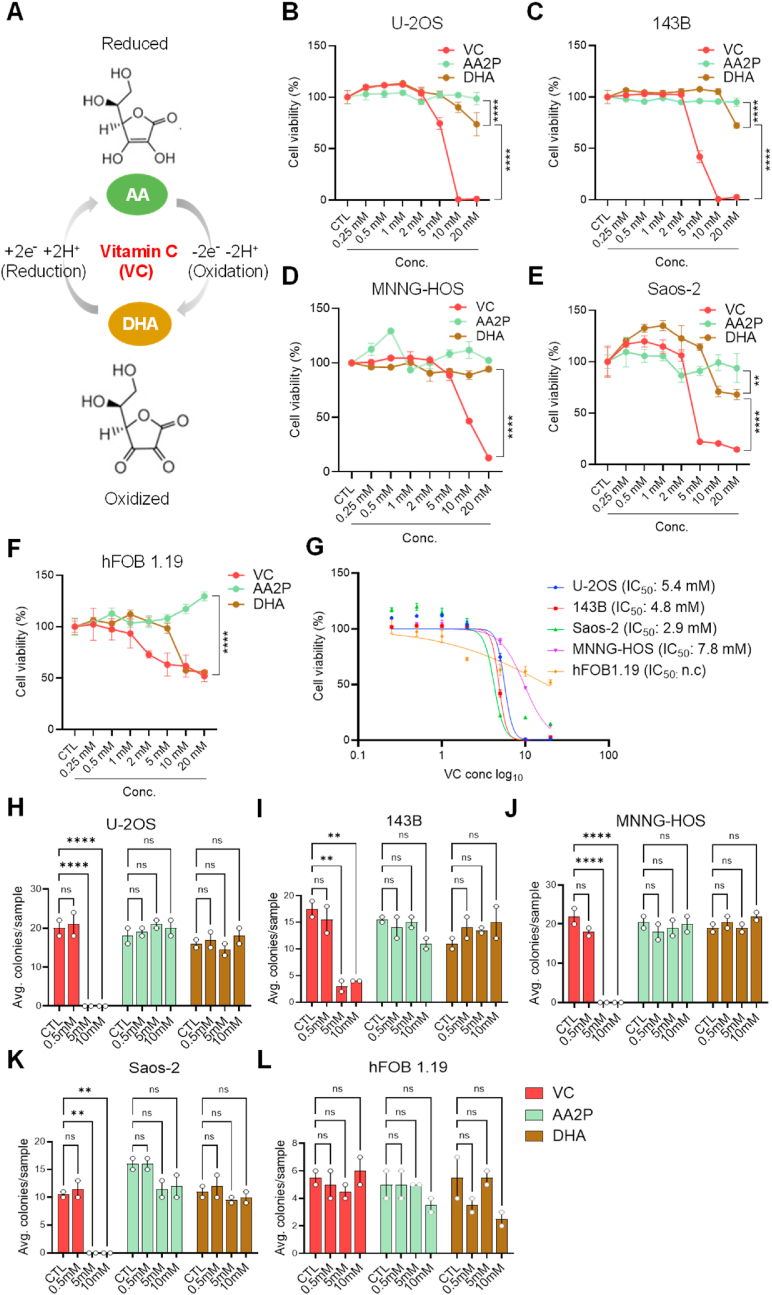
The oxidizable form of vitamin C induces cell death in human osteosarcoma cells at high doses. (**A**) Model of the interconversion between the reduced (ascorbic acid [AA]) and oxidized (dehydroascorbic acid [DHA]) forms of vitamin C (VC). (**B–F**) Alama blue cell viability analysis of (B) U–2OS, (C) 143B, (D) MNNG-HOS, (E) Saos-2, and (F) hFOB 1.19 cells after 24 h of treatment with different doses (0.25–20 mM) of VC (red), ascorbic acid 2-phosphate (AA2P, light green), or DHA (brown), as indicated. **p < 0.001, ****p < 0.0001 by one-way ANOVA. (**G**) Dose-response curves of the VC-dependent cytotoxicity in OS cells, as indicated. (**H-L**) Clonogenic survival assay of (H) U–2OS, (I) 143B, (J) MNNG-HOS, (K) Saos-2, and (L) hFOB 1.19 cells. The assay was conducted after 10 days of incubation of single cells trypsinized and seeded at a density of 200 cells/well in a 24-well plate, following 3 h of treatment with varying doses (0.5–10 mM) of VC (red), AA2P (light green), or DHA (brown), as indicated. See representative images associated with these data in Supplementary Fig. 1. **p < 0.001, ****p < 0.0001 by one-way ANOVA; ns, non significant. Data are representative of three independent experiments. (For interpretation of the references to color in this figure legend, the reader is referred to the Web version of this article.)

Physiologic VC exists largely in its reduced (ascorbic acid [AA]) or oxidized (dehydroascorbic acid [DHA]) forms, with dynamic interconversion between them, to act on diverse cellular functions and processes (Fig. 1A) [60]. In contrast to its antioxidant function at physiological levels, VC at high doses (1–10 mM) has been reported to increase ROS and preferentially kill cancer cells [9,61,62]. However, it is unclear which form of VC exerts this cytotoxic activity in tumors. Thus, to investigate the cancer cell-specific effects of different redox forms of VC, we subjected various OS cell lines (U–2OS, 143B, MNNG-HOS, Saos-2) derived from human patients as well as a human fetal osteoblastic cell line (hFOB 1.19), as the non-malignant control, to comparative cell viability analysis. After treating these cell lines with plain VC, ascorbic acid 2-phosphate (AA2P; a long-acting VC derivative that does not convert to DHA), or DHA at varying doses for 24 h, we found that plain VC at high doses (5–20 mM) markedly reduced cell viability in all the OS cell lines tested (U–2OS [IC_50_ = 5.4286 mM], 143B [IC_50_ = 4.8388 μM], MNNG-HOS [IC_50_ = 7.7813 μM], Saos-2 [IC_50_ = 2.9134 μM]), with much less of an effect on hFOB 1.19 control cells (Fig. 1B–G). Notably, DHA treatment at 10–20 mM resulted in slightly reduced cell viability in some OS cells (U–2OS, 143B, Saos-2) but not in MNNG-HOS cells or the hFOB 1.19 control cells, while AA2P treatment induced no changes in cell viability in any of the cell lines at similar doses (Fig. 1B–G). Corresponding to the results, the clonogenic survival assay showed that high doses of plain VC (5–10 mM), but not 0.5 mM, completely abrogated single-cell derived colony formation, with the high dose effect being significantly blunted in hFOB 1.19 cells, while AA2P and DHA did not exhibit such inhibitory effects at similar doses (Fig. 1H–L, Supplementary Fig. 1). These findings align well with previous studies indicating that high doses of VC may display cancer cell-specific toxicity while preserving normal cells [63]. We also found that this VC-induced cell death was significantly blocked by pretreatment with catalase, a key antioxidant enzyme that inhibits ROS-induced cell damage by catalyzing the decomposition of hydrogen peroxide (H_2_O_2_) into water and oxygen [64] (Supplementary Fig. 2A). In accordance with these results, using live-cell brightfield microscopy analysis, we observed noticeable cellular shrinkage and blebbing only in response to high-dose (5–20 mM) VC treatment (Supplementary Figs. 2B and C).

### 2.2 Pharmacological doses of oxidizable vitamin C impede the growth of 3D cultured human osteosarcoma spheroids

**Fig. 2:**
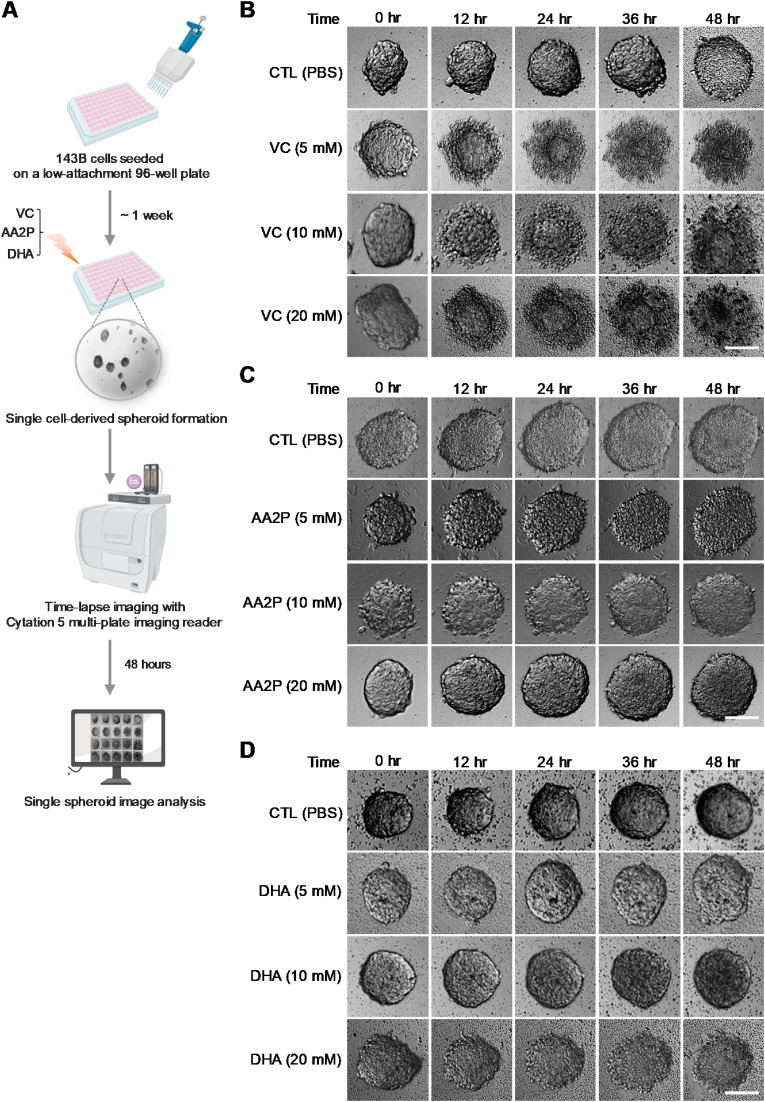
Pharmacological doses of oxidizable vitamin C inhibit the growth of 3D-cultured human OS spheroids. (**A**) Schematic diagram illustrating the procedure for time-lapse imaging analysis of 3D cultured human osteosarcoma (OS) spheroids For single cell-derived spheroid formation, 4 x 10^2^ 143B cells were cultured in 3D culture media in each well of a 96-well ultra-low attachment plate and incubated for one week. When detectable spheroids formed, time-lapse images were captured at 12-h intervals over a period of 48 h after treatment with vehicle (CTL), vitamin C (VC), AA2P, or DHA at the indicated doses (5–20 mM). Created with BioRender.com. (**B–D**) Representative time-lapse brightfield images showing the dose-dependent effects of VC (B), AA2P (C), and DHA (D), compared to PBS control (CTL), on the growth of OS spheroids. Data are representative of three independent experiments. Scale bars: 100 μm.

To further investigate the distinct effects of the VC isoforms on tumor growth, we utilized a three-dimensional (3D) spheroid culture system that closely mimics the native tumor environment (Fig. 2A). Interestingly, 143B cells showed the most efficient formation of spheroids from single cells under 3D culture conditions, compared to the other OS cell lines (Supplementary Figs. 3A and B). Similar to the findings in 2D monolayer cultures, the 3D 143B cell culture experiment showed that high dose VC (5–10 mM) completely abolished single-cell derived tumor spheroid formation though low-doses (0.125–0.5 mM) of VC had no significant effect on or tend to increase spheroid sizes and numbers (Supplementary Figs. 3C,D,E). Furthermore, time-lapse imaging analysis demonstrated that plain VC markedly inhibited tumor spheroid growth in 143B cell cultures in a dose- and time-dependent manner (Fig. 2B, Supplementary Fig. 4). Notably, treating spheroids with high-doses (5–20 mM) of VC led to severe cellular disintegration, with the generation of outer cell debris and the formation of residual spheroid bodies within 12 h (Fig. 2B). In contrast, treatment of 143B spheroids with high doses of AA2P or DHA did not result in similar inhibitory effects (Fig. 2C and D). Based on these findings, we hypothesized that the induction of OS cell death by VC primarily occurs through its oxidation process, resulting in acute generation of cytotoxic ROS upon treatment.

### 2.3 Pharmacological doses of oxidizable vitamin C induces cytotoxic ROS in human OS cells in an iron-dependent manner

**Fig. 3:**
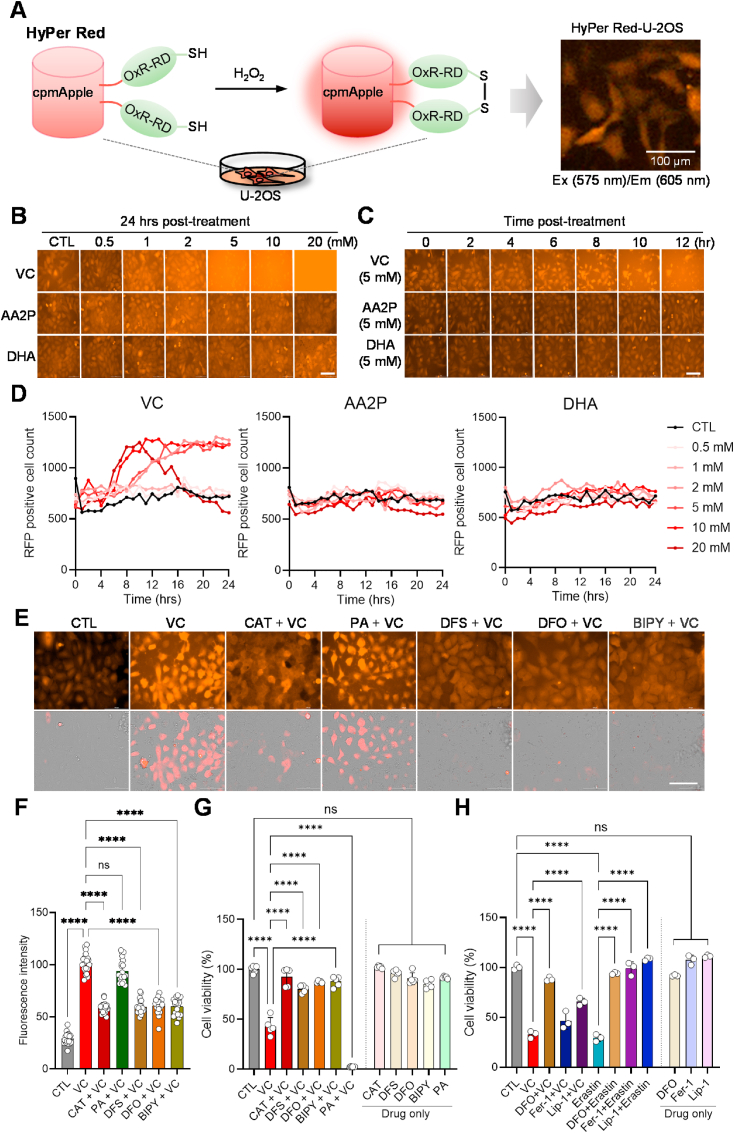
Oxidizable vitamin C induces cytotoxic ROS in human OS cells in an iron-dependent manner. (**A**) Illustration of intracellular hydrogen peroxide (H_2_O_2_) detection by the HyPer Red (cpmApple) probe in living cells. The OxyR-RD domain forms a reversible disulfide bond between cysteine residues, enabling OxyR to switch between its active and inactive states in response to the presence or absence of H_2_O_2_, respectively, within the cell. U–2OS cells stably expressing the HyPer Red probe were visualized with the red fluorescence filter set (excitation [Ex] 575 nm/emission [Em] 605 nm). (**B, C**) Dose- (B) and time- (C) dependent effects of VC, AA2P, and DHA on intracellular H_2_O_2_ levels, as indicated. Scale bars: 100 μm (**D**) Cell counting analysis of HyPer Red signal-positive U–2OS cells based on the imaging data presented in Fig. S4 showing the dose- and time-dependent effects of vitamin C, AA2P, and DHA on ROS generation. (**E**) Representative images of Hyper Red U–2OS cells in response to VC (5 mM) alone or in combination with iron chelators (Deferasirox [DFS, 100 μM], Deferoxamine [DFO, 100 μM], 2,2′-Bipyridyl [Bipy,100 μM]) or a copper chelator (Penicillamine [PA, 250 μM]). Cells were pretreated with the indicated drugs for 30 min before VC (5 mM) treatment for 5 h. Live-cell images were taken using the brightfield and red fluorescence filter cube (Ex. 531 nm/Em 593 nm) of a Cytation 5 multi-mode reader. The bottom images are presented with a brightfield merge. Scale bar: 100 μm (**F**) Quantitation of the fluorescence intensity of intracellular HyPer Red signal in individual cells (*n* = 25–30) treated with the drugs indicated in E. The original red fluorescent images of cells were converted to white and black mode, and the intensity of the signal was quantified using Image J software. (**G**) Cell viability analysis of U–2OS cells treated with VC (5 mM) alone or in combination with the indicated drugs for 24 h, as in E. (**H**) Cell viability analysis of U–2OS cells treated with VC (5 mM) or erastin (10 μM) alone or in combination with ferroptosis inhibitors (Deferoxamine [DFO, 100 μM], Ferrostatin-1 [Fer-1, 100 μM], Liproxstatin-1 [Lip-1, 0.5 μM]) for 24 h ***p < 0.0001 by two-way ANOVA with Tukey's multiple comparisons test; ns, non significant. The data show means normalized to untreated wells (Δ) ± SD of three independent experiments. (For interpretation of the references to color in this figure legend, the reader is referred to the Web version of this article.)

In order to further dissect the cellular mechanisms of the differential effects of VC derivatives, we stably introduced a plasmid encoding HyPer Red (cpmApple), a genetically modified fluorescent indicator that detects intracellular H_2_O_2_ levels via specific sensor domains (OxyR-RD) [65], into U–2OS cells (Fig. 3A). Live-cell imaging analysis of these cells revealed that treatment with plain VC, but not AA2P or DHA, dramatically increased intracellular and extracellular H_2_O_2_ levels in a dose-dependent manner (Fig. 3B). Moreover, time-lapse imaging analysis revealed that at high doses of plain VC (5–20 mM), the cells began to shrink and showed blebbing, with the loss of HyPer Red signal within a few hours, whereas treatment with AA2P or DHA did not affect intracellular ROS levels or cellular morphology over the time course of the experiment (Fig. 3C and D, Supplementary Fig. 5). These results nicely correspond to the cell viability assay results shown in Fig. 1.

Transition metals, such as copper (Cu^2+^) and iron (Fe^3+^/Fe^2+^), have been implicated in the oxidation of AA to DHA through the Fenton reaction, leading to the generation of H_2_O_2_ [66]. To investigate the role of transition metals in VC-induced ROS production in OS cells, we treated our HyPer Red indicator cells with penicillamine (PA), a copper chelating agent used in the treatment of Wilson's disease [67], or various iron chelators (deferasirox [DFS], deferoxamine [DFO], and 2,2′-Bipyridyl [Bipy]) prior to VC treatment. Live-cell imaging analysis revealed that the iron chelators (DFS, DFO, and Bipy) effectively reduced the VC-induced H_2_O_2_ levels to a similar extent as catalase, whereas pretreatment with the copper chelator (PA) did not exhibit such an inhibitory effect (Fig. 3E and F). In line with these results, subsequent cell viability assays showed that pretreatment with the iron chelators significantly prevented VC-induced cell death, whereas pretreatment with the copper chelator (PA) had no such effect (Fig. 3G, left). Importantly, these drugs had minimal effects on cell viability when administered alone (Fig. 3G, right). These aligns with a recent report intracellular excess labile iron increases pharmacological ascorbate-mediated oxidative stress by Fenton reaction in OS cell lines (MG63, MNNG/HOS, U–2OS) [11]. These results indicate that iron plays a crucial role in ROS-associated cytotoxicity following VC treatment.

Ferroptosis has been suggested as an iron-dependent form of cancer cell death [68]. To explore the potential involvement of ferroptosis in the cytotoxic effects of VC, we treated U–2OS cells with specific ferroptosis inhibitors (ferrostatin-1 [Fer-1, 100 μM], liproxstatin-1 [Lip-1, 0.5 μM], before treating with VC (5 mM) or erastin (10 mM), a specific ferroptosis activator (Fig. 3H). Interestingly, unlike the iron chelator (DFO), the ferroptosis inhibitor Fer-1 did not provide significant protection, and Lip-1 only partially protected against VC-induced cell death, although they completely inhibited erastin-induced cell death. (Fig. 3H). Consistent with this result, a recent report showed that these ferroptosis inhibitors are ineffective at protecting against cell death in N-RAS-mutant HT-1080 fibrosarcoma and non-RAS-mutant MCF-7 breast cancer cell lines under lethal doses of pharmacological ascorbate [69]. These findings indicate that iron-dependent cell death induced by VC treatment involves additional mechanisms beyond ferroptosis.

### 2.4 Pharmacological vitamin C induces non-apoptotic cell death in human OS cells in an intracellular Ca^2+^-dependent manner

Ascorbic acid has been reported to display anti-tumor effects by acting as a cofactor for diverse Fe^2+^- and 2-oxoglutarate-dependent dioxygenases, including hypoxia-inducible factor (HIF) hydroxylases (PHDs 1–3 and FIH) and DNA demethylases (TET1-3) [60] (Supplementary Fig. 6A). In addition, it has been reported that VC-induced cell death occurs via the activation of multiple cell death (e.g., apoptosis, necroptosis, necrosis) pathways [44]. Notably, calcium (Ca^2+^) plays a crucial role in mediating both apoptotic and non-apoptotic forms of cell death triggered by ROS-inducing anti-cancer agents [[50], [51], [52]]. To explore the involvement of these pathways in VC-induced OS cell death, we conducted a comparative cell viability analysis using the pathway-specific inhibitors BAPTA AM, a membrane-permeable intracellular calcium inhibitor (BAPTA, 10 μM); Bobcat339, a DNA methyltransferase inhibitor (Bobcat, 33 μM); dimethylallyl glycine, a HIF inactivator (DMOG, 2 μM); Z-VAD-FMK, a pan-caspase inhibitor (ZVAD, 10 μM), and necrostatin-1, a necroptosis inhibitor (Nec-1,10 μM). U–2OS cells were treated with inhibitor either alone or in combination with VC (5 mM) for 24 h before measuring cell viability. Interestingly, the results revealed that the membrane-permeable Ca^2+^ chelator (BAPTA) completely blocked VC-induced cell death, similar to catalase (CAT), while none of the other inhibitors were able to rescue VC-mediated cell death (Supplementary Fig. 6B). Concomitantly, live-cell imaging experiments using HyPer Red U–2OS cells showed that, similar to CAT, BAPTA effectively reduced VC-induced ROS generation compared to the other drugs (Supplementary Figs. 6C and D). Moreover, treatment with an exogenous calcium chelator (EGTA) did not efficiently block VC-induced ROS production and cell death, indicating that intracellular calcium pathways play a more prominent role than extracellular calcium pathways in VC-induced cytotoxicity (Supplementary Fig. 7). Alternatively, the divergent outcomes could be attributed to a slower rate or potential nonspecific binding of EGTA to other metals, compared to BAPTA, in capturing Ca^2+^ ions [70,71]. Notably, neither individual nor combined application of ZVAD and Nec1 were protective against VC-induced ROS production and cell death (Supplementary Fig. 6B, Supplementary Fig. 7). Consistent with these results, live-cell fluorescence recording and imaging analyses performed using SYTOX necrosis and Annexin V Cy3 apoptosis staining dyes indicated that, unlike pretreatment with CAT or BAPTA, neither ZVAD nor Nec1 pretreatment prevented VC-induced apoptosis or necrosis in U–2OS cells (Supplementary Figs. 6C and D). Similar to our findings, recent studies have reported that Nec1 and ZVAD do not inhibit pharmacologic VC-induced cell death in other types of cancer cells [34,49]. Consistent with the results in 2D monolayer cultures, the 3D 143B cell culture experiment showed that BABTA pretreatment markedly inhibited the high dose VC (5 mM)-induced suppression in single-cell derived OS spheroid formation, resulting in spheroid sizes and numbers similar to control or BAPTA-only treated samples (Supplementary Figs. 6E–G). These results suggest that intracellular Ca^2+^ pathways are critical for VC-induced OS cell death via non-canonical apoptotic mechanisms [72].

### 2.5 Pharmacological vitamin C induces human OS cell death by triggering Ca^2+^ release through IP3Rs in the ER and mitochondrial ROS

**Fig. 4:**
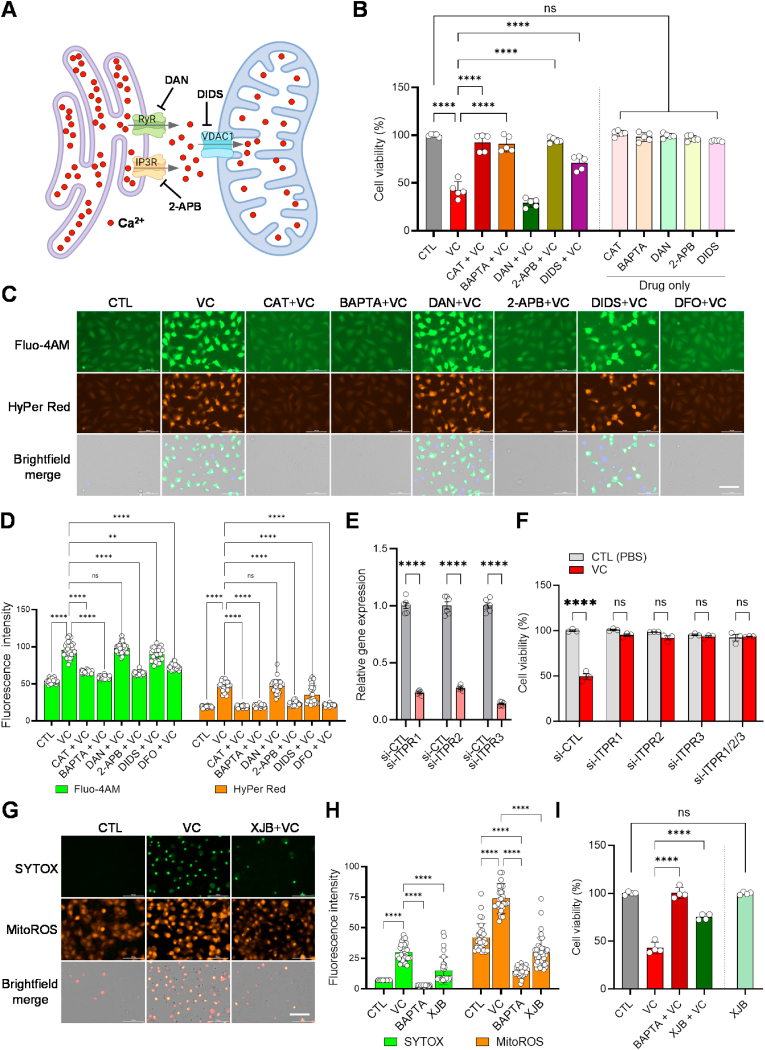
Vitamin C induces cell death by triggering Ca^2+^ release through IP3Rs in the ER and mitochondrial ROS. (**A**) Schematic diagram illustrating pharmacological inhibition of Ca^2+^ release channels (inositol 1,4,5-triphosphate receptor [IP3R/ITPR], ryanodine receptor [RyR]) in the ER and the Ca^2+^ influx channel voltage-dependent anion channel 1 (VDAC1) on mitochondria. Created with BioRender.com. (**B**) Cell viability analysis of U–2OS cells treated with vitamin C (VC, 5 mM] and/or drugs to block the movement of calcium (Catalase [CAT, 2000u], BAPTA AM intracellular calcium inhibitor [BAPTA, 10 μM], Dantrolene [DAN, 60 μM], 2-Aminoethoxydiphenyl borate [2-APB, 100 μM], 4,4′-Diisothiocyano-2,2′-stilbenedisulfonic acid [DIDS, 100 μM]), as indicated, for 24 h ****p < 0.0001 by one-way ANOVA with Tukey's multiple comparisons test; ns, non-significant. The data show means normalized to untreated wells (Δ) ± SD of three independent experiments. (**C**) Representative live-cell images of HyPer Red reporter U–2OS cells loaded with Fluo-4AM calcium indicator following exposure to VC (2 mM) and the indicated drugs alone or in combination (as in A) for 6 h. Images were taken using specific fluorescence filter sets for the detection of Fluo-4AM green dye (Ex. 488 nm/Em 530 nm) and Hyper-Red (Ex. 543 nm/Em 570 nm) fluorescence signal. Scale bars: 100 μm (**D**) Quantitation of the intracellular Fluo-4AM and HyPer Red signals in individual cells (*n* = 25–30) treated with the indicated drugs, as shown in C. Original green or red fluorescent images of cells were converted to white and black mode, and signal intensities were quantified using Image J. (**E**) qPCR analysis to verify the efficiency of siRNAs targeting the indicated *ITPR* isoforms. (**F**) The effects of knocking down *ITPR* family genes on high-dose vitamin C-induced cytotoxicity in U–2OS cells are shown. Forty-eight hours after transfection with control siRNA (si-CTL) or siRNAs targeting *ITPR* isoforms alone or in combination, as indicated, cells were exposed to 5 mM VC for 24 h and subsequently subjected to cell viability assays. Statistical analysis using two-way ANOVA and Tukey's multiple comparisons test revealed significant differences (****p < 0.0001); ns, non-significant. Data representative of three independent experiments are shown as means ± SD (*n* = 3). (**G**) Representative images of cells loaded with SYTOX necrosis and MitoROS mitochondrial ROS staining dyes following exposure to VC (5 mM) alone or in combination with BAPTA (10 μM) or the mitochondria-targeting antioxidant XJB-5-131 [XJB, 60 μM] for 5 h. Specific fluorescence filter sets were used for the detection of SYTOX Green (Ex. 488 nm/Em 530 nm) and MitoROS (Ex. 543 nm/Em 570 nm). Scale bars: 100 μm. (**H**) Quantitation of intracellular SYTOX and MitoROS signals in individual cells (*n* = 25–30) treated with the indicated drugs using Image J, as described in D. (**I**) Cell viability assays were performed in U–2OS cells treated with PBS (CTL) or VC (5 mM) alone or in combination with BAPTA (10 μM) or XJB (60 μM) for 24 h ****p < 0.0001 by one-way ANOVA with Tukey's multiple comparisons test; ns, non-significant. Data are representative of three independent experiments. (For interpretation of the references to color in this figure legend, the reader is referred to the Web version of this article.)

The ER acts as the primary intracellular Ca^2+^ reservoir, regulating cytosolic Ca^2+^ levels and releasing Ca^2+^ via inositol 1,4,5-triphosphate receptors (IP3Rs) and/or ryanodine receptors (RyRs) upon stimulation to modulate various cellular responses, such as proliferation and cell death [73]. In addition, the release and subsequent overload of Ca^2+^ into mitochondria via the voltage-dependent anion channels (VDACs) have been implicated in promoting cell death in response to cytotoxic stimuli, including oxidative stress induced by anti-cancer agents [74] (Fig. 4A). Using Fluo-4AM, an intracellular calcium indicator and Rhod-2AM, a mitochondrial calcium indicator, in live-cell Ca^2+^ imaging analysis of U–2OS cells, we observed significantly increased fluorescent signals for both indicators after exposure to VC (5 mM), compared to the control (Supplementary Fig. 8). To investigate the involvement of ER-mitochondrial calcium transport channels in VC-induced cell death, we pre-treated U–2OS cells with specific IP3R (2-aminoethoxydiphenyl borate [2-APB]), RyR (dantrolene [DAN]), and VDAC (4,4′-diisothiocyanostilbene-2,2′-disulfonic acid [DIDS]) inhibitors prior to VC treatment. Interestingly, pretreatment with the IP3R blocker (2-APB) effectively prevented VC-induced cell death, similar to the effects observed with CAT and BAPTA (Fig. 4B). However, pretreatment with the RyR blocker (DAN) did not show any protective effects, while treatment with the VDAC blocker (DIDS) partially rescued VC-induced cytotoxicity (Fig. 4B). These results were recapitulated by further clonogenic cell survival assay showing that 2-APB, but not DAN, significantly blocked VC-suppressed single cell-derived colony formation to a comparable level with CAT, DFO, and BAPTA, with a relatively moderate rescue effect observed with DIDS (Supplementary Figs. 9A and B). In line with these results, live-cell Ca^2+^ imaging analysis using HyPer Red U–2OS cells loaded with Fluo-4AM demonstrated that, similar to CAT, BAPTA and DFO, 2-APB significantly reduced both calcium and H_2_O_2_ levels induced by VC treatment, whereas DAN did not have a significant effect, and DIDS showed only a partial inhibitory effect (Fig. 4C and D). In accordance with the pharmacological results, genetic perturbation of genes encoding IP3R isoforms (*ITPR1*, *ITPR2*, *ITPR3*) via RNA interference (RNAi), individually or in combination, significantly rescued VC-induced cell death and suppression of single-driven colony formation in both U–2OS and 143B cells (Fig. 4E and F, Supplementary Figs. 10A–C). Endoplasmic reticulum oxidoreductase 1 alpha (ERO1α) has been reported to stimulate IP3R activity in calcium-dependent apoptosis under ER stress [75]. Similar to the ITPR RNAi results, further cell viability and clonogenic cell survival assays showed that knockdown of gene (*ERO1A*) encoding ERO1α significantly prevented VC-induced cell death and mitigated the suppression of colony formation in the human OS cells (Supplementary Figs. 10D–F). Notably, the rescue effect was more pronounced in cell viability assays, suggesting higher VC cytotoxic sensitivity at the single-cell level. These results suggest that IP3Rs serve as the primary channels responsible for the release of Ca^2+^ from the ER upon VC treatment, resulting in the subsequent lethal outcomes.

Previous studies have suggested that Ca^2+^ influx and mitochondrial ROS production are closely linked in mediating cell death [76]. To determine the role of mitochondrial ROS in VC-induced cell death, we treated U–2OS cells with XJB-5-131 (XJB, 60 μM), a mitochondria-targeted ROS and electron scavenger [77], prior to VC (5 mM) administration. Live-cell dual fluorescence imaging analysis using SYTOX and mitoROS, a mitochondrial ROS indicator, demonstrated that XJB effectively reduced VC-induced necrosis and mitochondrial ROS signals to levels similar to those of untreated control cells; however, its inhibitory effect was less pronounced than what was seen with BAPTA (Fig. 4G and H). Cell viability and clonogenic cell survival assays also showed that XJB provided significant protection against VC-induced cell death and suppression of the colony formation, although still not to the same extent as BAPTA (Fig. 4I, Supplementary Figs. 9A and B). These findings indicate that, along with ROS generated through the iron-dependent Fenton reaction, mitochondrial ROS production resulting from Ca^2+^ influx plays an important role in VC-induced cell death in OS cells.

### 2.6 Pharmacological vitamin C impacts the expression of mitochondrial respiratory genes

**Fig. 5:**
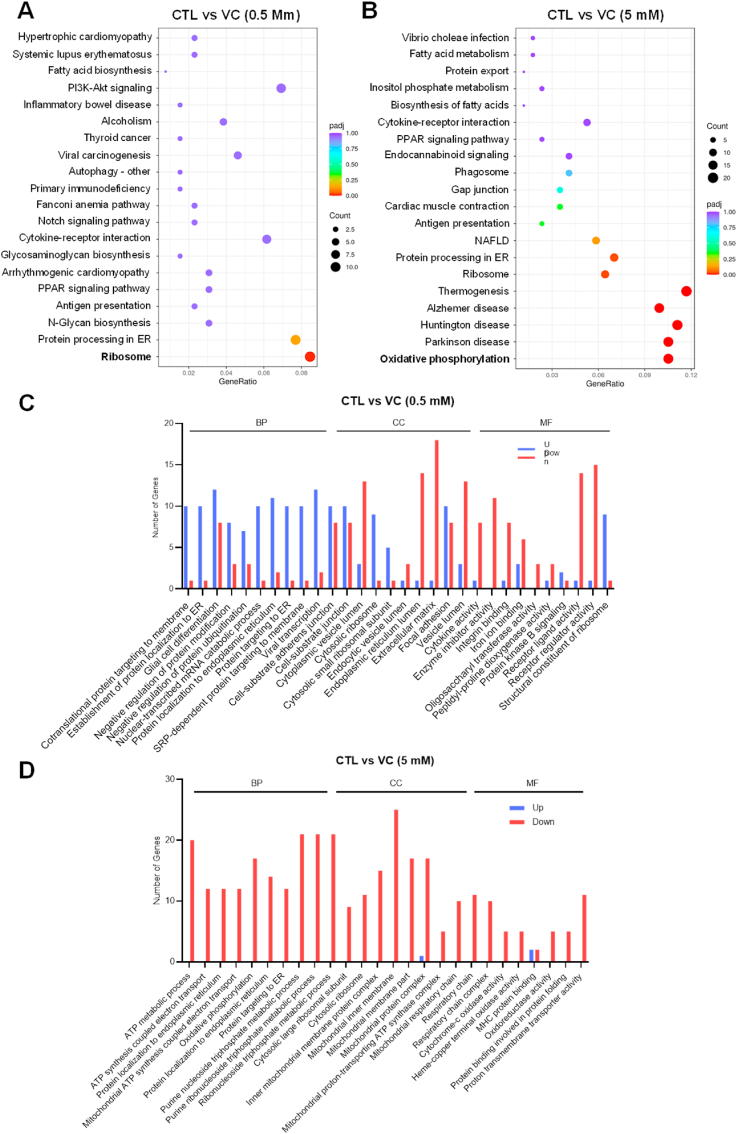
GO and KEGG pathway enrichment analyses of differentially expressed mRNAs in U–2OS cells treated with vitamin C. (**A, B**) Dot plots showing the results of Gene Ontology (GO) and Kyoto Encyclopedia of Genes and Genomes (KEGG) pathway enrichment analyses performed on genes differentially expressed in control and VC-treated (0.5 mM (A) and 5 mM. (B)) U–2OS cells. Plot colors represent the adjusted *p*-values (padj), and sizes represent gene numbers. (**C, D**) Bar plots showing the number of up-regulated (Up, blue) and down-regulated (Down, red) genes in the significant GO terms (FDR <0.05) identified by the GO analyses of the genes differentially expressed with VC treatment (0.5 mM (C) and 5 mM (D)). CC = cellular component, BP = biological process, MF = molecular function. (For interpretation of the references to color in this figure legend, the reader is referred to the Web version of this article.)

**Fig. 6:**
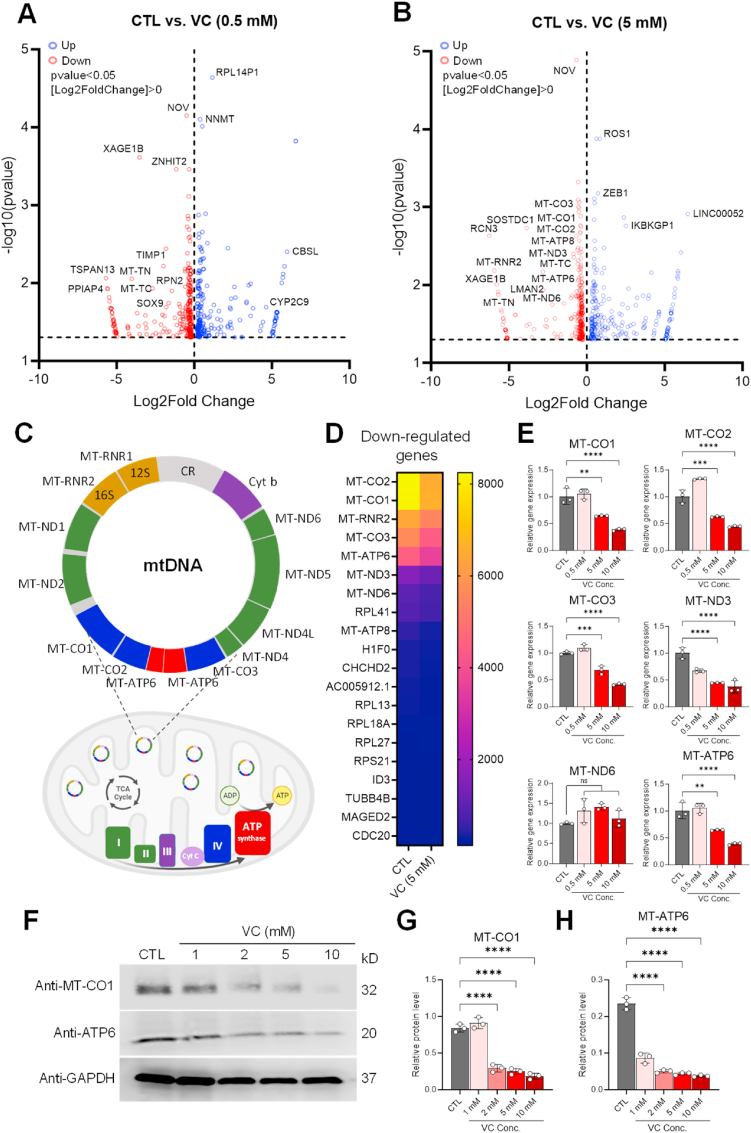
Pharmacological vitamin C reduces mitochondrial respiratory gene expression. (**A, B**) Volcano plots showing the genes down-regulated (Down, red) and up-regulated (Up, blue) in U–2OS cells following treatment with low- (0.5 mM) (C) and high- (0.5 mM) (D) dose vitamin C (VC). (**C**) A schematic representation of mitochondrial DNA (mtDNA) and the mitochondrial respiratory chain. The colors of the genes on the mtDNA molecule correspond to their cognate subunits of the electron transport chain: six subunits of NADH dehydrogenase (MT-ND 1–6) of complex I (green), three subunits of cytochrome *c* oxidase (MT-CO1∼3) of complex IV (blue), the cytochrome *b* subunit (Cyt *b*) of complex III (purple), and two subunits of adenosine triphosphate (ATP) synthase (MT-ATP6 and MT-ATP8; red). 12S and 16S ribosomal RNAs (e.g., MT-RNR1, MT-RNR2) are shown in dark yellow. The control region (CR) controls the initiation of replication and transcription of mtDNA. ND, NADH dehydrogenase, CO, cytochrome *c* oxidase; Cyt *b*, cytochrome *b*; RNR, Ribosomal RNA gene. (**D**) Heat map of the mitochondrial genes that were down-regulated in VC (5 mM)-treated cells relative to control (CTL) cells. (**E**) qPCR analysis of the dose-dependent effects of VC (0.5 mM, 5 mM, 10 mM) on the mRNA expressions of representative mitochondrial respiratory genes, as indicated. (**F**) Western blot analysis of the dose-dependent effects of VC (1–10 mM) on the protein expressions of MT-CO1 and MT-ATP6. (**G, H**) Densitometry analysis of the Western blot data shown in F. Data were normalized to GAPDH and are represented as means ± SEM from *n* = 3 independent experiments. (For interpretation of the references to color in this figure legend, the reader is referred to the Web version of this article.)

To gain further insights into the molecular impacts of VC treatment, we conducted RNA sequencing (RNA-seq) analysis on U–2OS cells either untreated or treated with low (0.5 mM) or high (5 mM) concentrations of VC (Supplementary Fig. 11A, Supplementary Materials). Subsequent differential expression analysis comparing untreated control and VC-treated cells identified a total of 417 differentially expressed genes (DEGs, p < 0.05) (199 down-regulated and 218 up-regulated) in cells treated with the low concentration of VC and 480 DEGs (249 down-regulated and 231 up-regulated) in cells treated with the high concentration of VC (Supplementary Figs. 11B and C). Interestingly, we observed that 30.1 % (60/199) of the genes that were down-regulated by the low concentration of VC were also down-regulated by the high concentration of VC, indicating shared gene expression patterns (Supplementary Fig. 11B). Similarly, 22.9 % (50/218) of the genes up-regulated in response to the low dose of VC were also found among the subset of genes up-regulated in response to the high dose of VC (Supplementary Fig. 11C). Gene Ontology (GO) and Kyoto Encyclopedia of Genes and Genomes (KEGG) pathway enrichment analyses of the DEGs revealed that low-dose VC treatment primarily affected cellular pathways related to protein processing in the ER and ribosomes (Fig. 5A). On the other hand, high-dose VC treatment had the most impact on pathways related to oxidative phosphorylation (Fig. 5B). Further KEGG pathway analysis revealed that DEGs in cells treated with low-dose VC exhibited diverse gene expression profiles associated with the processing of proteins in the ER as well as ribosome-related pathways (Fig. 5C). In contrast, cells treated with high-dose VC showed substantial down-regulation of a large set of genes associated with mitochondrial metabolic pathways (Fig. 5D). Interestingly, subsequent volcano plot analysis revealed that several of the genes that were down-regulated with high-dose VC treatment were from the mitochondrial genome, including *MT-CO1-3*, *MT-ATP6*, *MT-ATP8*, *MT-ND3*, and *MT-ND6* (Fig. 6B–D). These genes encode molecular components of the electron transport chain (ETC), which play a role in mitochondrial OXPHOS and ATP synthesis (Fig. 6C). Only two genes from the mitochondrial genome, the transfer RNA genes *MT-TN* and *MT-TC*, were down-regulated in response to treatment with low-dose VC (Fig. 6A). Notably, the overall expression of glycolysis-related genes, which are derived from the nuclear genome, remained unaffected by exposure to high-dose VC (Supplementary Fig. 12).

To validate the RNA-seq results, we conducted quantitative reverse transcription-PCR (qRT-PCR) analysis on six specific mitochondrial genes to confirm their mRNA levels in OS cells with and without VC treatment. Consistent with the RNA-seq data, the qRT-PCR results demonstrated significantly down-regulated expression of five genes encoding ETC subunits of complex I (*MT-ND3*), complex IV (*MT-CO1*, *MT-CO2*, *MT-CO3*), and ATP synthase (*MT-ATP6*) in response to high-dose VC (0.5–10 mM) (Fig. 6E). Subsequent Western blot analysis further confirmed the dose-dependent down-regulation of MT-CO1 and MT-ATP6 protein expression (Fig. 6F–H, Supplementary Fig. 13). These data suggest that high-dose VC impacts mitochondrial genome integrity and function related to cellular bioenergetics.

### 2.7 Pharmacological vitamin C disrupts mitochondrial functions and energy metabolism in human OS cells

**Fig. 7:**
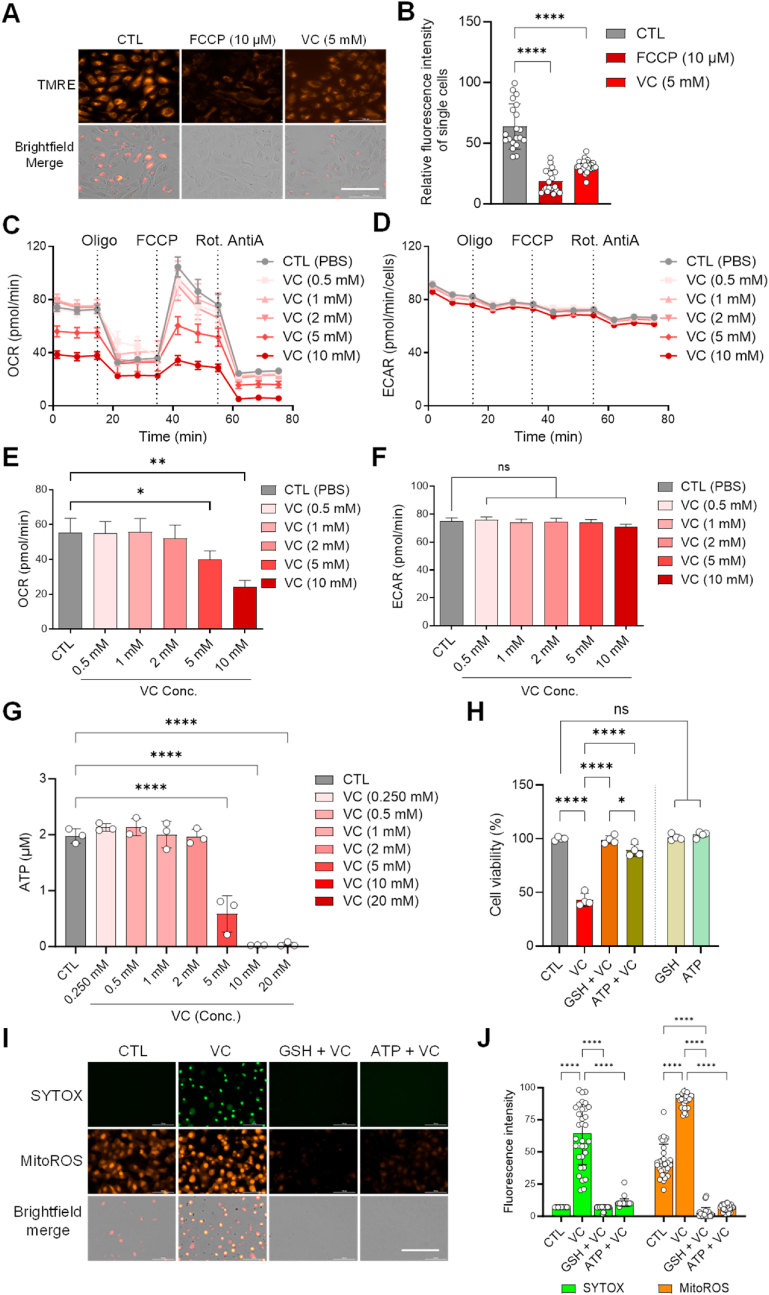
High-dose vitamin C disrupts mitochondrial functions and energy metabolism to induce cell death in OS cells. (**A**) U–2OS cells were seeded in 96-well plates and grown to about 70 % confluence. Cells were then treated with PBS, FCCP (10 μM), or VC (5 mM) and stained with TMRE (tetramethylrhodamine, ethyl ester) dye at 25 nM. Images were captured using a BioTek Cytation 5 multi-mode imaging plate reader at 20X with the RFP and brightfield filter sets. (**B**) The fluorescence intensity of TMRE per cell (*n* > 20 cells) was quantified using Image J software. TMRE is used to label active mitochondria. (**C–F**) Metabolic flux analysis of the oxygen consumption rate (OCR) and extracellular acidification rate (ECAR) in U–2OS cells treated with or without VC. The OCR (C, E) and ECAR (D, F) were monitored to determine the dose-dependent effects of VC (0.5–10 mM) on mitochondrial respiration and glycolysis. For the OCR assays, all cells were exposed sequentially to oligomycin, FCCP, and rotenone plus antimycin A. *p < 0.05, **p < 0.005 by one-way ANOVA with Tukey's multiple comparisons test; ns, non-significant. Error bars indicate standard error of the mean (SEM), *n* = 6 per group. (**G**) ATP detection assays were performed in U–2OS cells treated with PBS or VC at the indicated concentrations for 6 h (0.25–20 mM). (**H**) Cell viability assays were conducted in U–2OS cells pretreated with GSH (100 μM) or ATP (100 μM) for 1 h and then treated with PBS (CTL) or VC (5 mM), as indicated, for 24 h *p < 0.05, ****p < 0.0001 by two-way ANOVA with Tukey's multiple comparisons test; ns, non-significant. (**I**) Representative images of cells loaded with SYTOX necrosis and MitoROS mitochondrial ROS staining dyes following exposure to VC (5 mM) alone or in combination with the indicated drugs for 5 h. Specific fluorescence filter sets were used for the detection of SYTOX Green (Ex. 488 nm/Em 530 nm) and MitoROS (Ex. 543 nm/Em 570 nm). (**J**) Quantitation of the intracellular SYTOX and MitoROS signals in individual cells (*n* = 25–30) treated with the indicated drugs, as shown in (I). The original green and red fluorescent images of cells were converted to white and black mode, and the intensity of the signal was quantified using Image J. ****p < 0.0001 by two-way ANOVA with Tukey's multiple comparisons test; ns, non-significant. Data are representative of three independent experiments. (For interpretation of the references to color in this figure legend, the reader is referred to the Web version of this article.)

Mitochondrial membrane potential (MMP, ΔΨm) is known as a key indicator of mitochondrial activity because it reflects the process of electron transport by proton pumps (Complexes I, III and IV) and OXPHOS, the driving force behind ATP production [78]. MMP assays using tetramethylrhodamine, ethyl ester (TMRE), a red-orange fluorescent dye capable of detecting active mitochondria, showed that high-dose VC (5 mM) significantly reduced MMP to levels similar to those induced by FCCP, a positive control for mitochondrial membrane depolarization (Fig. 7A and B). In addition, real-time metabolic flux analysis (MFA) revealed that the oxygen consumption rate (OCR), a marker of mitochondrial OXPHOS, was significantly reduced with 5–10 mM VC treatment in both U–2OS and 143B cells (Fig. 7C–E, Supplementary Figs. 14A and C). However, a dose-dependent decrease in extracellular acidification rate (ECAR), which serves as an indicator of glycolysis, was not evident in concurrent measurements (Fig. 7D–F, Supplementary Figs. 14B and D). Consistent with this, measurements of ATP showed marked reductions in ATP levels with high-dose VC treatment (5–20 mM), whereas no significant changes in ATP levels were observed with low-dose VC treatment (Fig. 7G, Supplementary Fig. 14E). These results further support the impact of high-dose VC on mitochondrial ATP production during VC-induced cell death. In line with this notion, pretreatment with ATP reduced the cell death and single cell-derived colony formation induced by high-dose VC in a manner comparable to treatment with glutathione (GSH), which is also known to be depleted by VC treatment and/or inhibit VC-induced cytotoxicity [54] (Fig. 7H, Supplementary Figs. 9A and B). Additionally, in dual fluorescence imaging analysis of cells co-labeled with SYTOX and mitoROS indicators, ATP pretreatment was found to effectively inhibit both necrotic cell death and the production of mitochondrial ROS induced by VC (Fig. 7I and J). Given the important role of mitochondrial ETC subunits in ATP production via oxidative phosphorylation, these findings collectively suggest that the loss of ATP production via mitochondrial respiratory dysfunction is critical for the process of high-dose VC-induced cell death in OS cells.

### 2.8 Pharmacological vitamin C reduces tumor growth in the orthotopic model of human osteosarcoma through mitochondrial damage

**Fig. 8:**
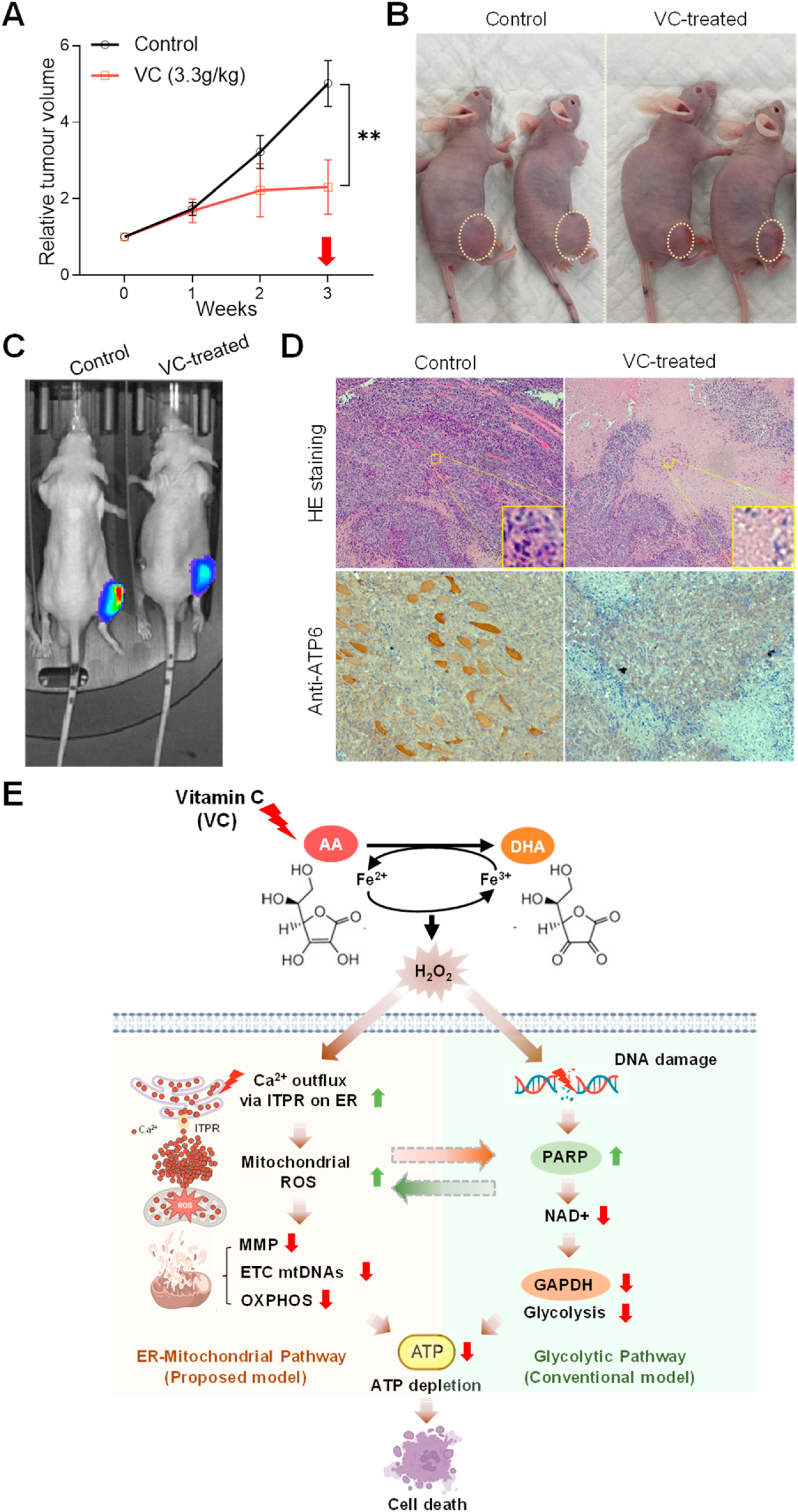
High-dose vitamin C reduces tumor growth in the orthotopic model of human osteosarcoma. (**A**) After orthotopic injection of a 143B-Luc osteosarcoma cell suspension (approximately 1 x 10^6^ cells) into the right tibia, mice were intraperitoneally administered either a vehicle (water, *n* = 6) or vitamin C (3.3 g/kg) (VC, *n* = 6) solution on a daily basis once detectable tumors were formed. Tumor volumes (mm^3^) in both control and VC-treated mice were measured weekly for three weeks using a digital caliper. The red arrow indicates the final IVIS bioluminescence imaging and sacrifice for subsequent analysis. **p < 0.005, determined by two-way ANOVA and Tukey's multiple comparisons test. Data are represented as means ± SEM. (**B**) Representative images of xenografted OS tumors in control and VC-treated mice. Yellow dashed circles indicate the tumors measured. (**C**) Representative results of IVIS bioluminescence imaging of orthotopic tumor growth in control and VC-treated mice at the third week post-vehicle/VC administration. (**D**) Representative images of tumor tissues harvested after 3 weeks of treatment with vehicle or VC that were stained with hematoxylin and eosin (HE) (upper panels) or subjected to immunohistochemistry (IHC) analysis with anti-MT-ATP6 antibody (lower panels). (**E**) Potential mechanism of VC-induced cancer cell death via metabolic crosstalk between dysregulated ER-mitochondrial and glycolysis pathways**.** The proposed model shows that the ER-mitochondrial and glycolysis pathways may interact synergistically to induce an energy crisis and cell death in response to VC treatment. VC generates H_2_O_2_ via its oxidation from AA to DHA in an iron-dependent manner. On the one hand, H_2_O_2_ triggers Ca^2+^ release from the ER via ITPR, which, in turn, increases mitochondrial calcium and ROS and is accompanied by decreased MMP and reduced expression of mitochondrial genes related to OXPHOS, eventually leading to ATP depletion and cell death. On the other hand, H_2_O_2_ causes DNA damage, leading to enhanced poly(ADP-ribose) polymerase (PARP) activation, which may consume NAD and deplete ATP through reduced glyceraldehyde 3-phosphate dehydrogenase (GAPDH) activity and glycolysis. ITPR = inositol 1,4,5-trisphosphate receptor, OXPHOS = oxidative phosphorylation, ETC = Electron transport chain, MMP = mitochondrial membrane potential, mtDNA = mitochondrial DNA, GAPDH = glyceraldehyde-3-phosphate dehydrogenase. (For interpretation of the references to color in this figure legend, the reader is referred to the Web version of this article.)

To assess the impact of high-dose VC on OS development *in vivo*, we generated 143B-luciferase OS cells (143B-Luc) and orthotopically injected them into the right tibias of mice. Following detectable tumor formation, mice were intraperitoneally administered a daily solution of either vehicle (water, control) or VC (3.3 g/kg) (VC-treated). Tumor volumes were then measured weekly for three weeks using a digital caliper. As anticipated, we observed a significant reduction in the tumor growth rate in VC-treated mice compared to vehicle-treated mice (Fig. 8A and B). *In vivo* bioluminescence (IVIS) imaging analysis further demonstrated decreased luciferase activity in the tumors of VC-treated mice compared to tumors from control mice (Fig. 8C). Subsequent hematoxylin and eosin (H&E) staining of OS tumor sections revealed that control group tissues exhibited intact tumor cell structures with a distinct nucleus in each individual cell (Fig. 8D, Supplementary Fig. 15A). However, this well-defined staining pattern was substantially lost in tumors treated with high-dose VC (Fig. 8D, Supplementary Fig. 15A). Moreover, immunohistochemistry (IHC) analysis showed markedly reduced staining for mitochondrial ATP synthase (MT-ATP6) expression in VC-treated mouse tumor tissues compared to control tumor tissues, consistent with our *in vitro* results (Fig. 6, Fig. 8D, Supplementary Fig. 15B). Collectively, these data suggest that high-dose vitamin C negatively impacts OS tumor growth via mitochondrial damage mechanisms *in vivo*.

## 3 Discussion

The role of vitamin C in cancer has been a matter of debate due to the complex interplay between its reduced (AA) and oxidized (DHA) forms under varying physiological conditions. According to the prevailing model, DHA is considered the most potent pharmacological form of vitamin C, as it generates ROS upon conversion to AA [17,18]. However, recent studies using DHA directly have consistently shown that this form has minor or no significant effect on cell death in multiple cancer cell lines, including human breast cancer and neuroblastoma cells [20,21,79]. In addition, a growing number of studies have shown that AA2P, a long-acting derivative of VC, exhibits antioxidant properties in rescuing both normal and cancer cells, including renal cancer cells and human SH-SY5Y neuroblastoma cells, from cell death when used alone or in combination with ROS-inducing or scavenging agents at low to moderate concentrations (0.2–0.8 mM) [[80], [81], [82], [83]]. These findings align with our 2D and 3D OS model data showing that the natural form of VC exhibits greater cytotoxicity against various human OS cell types than DHA or AA2P when used at similarly high concentrations (5–20 mM) (Fig. 1, Fig. 2, Fig. 3). Furthermore, our pharmacological data show that iron, but not copper, is mainly responsible for VC-induced ROS generation (Fig. 3E). These results coincide with a recent biomolecular study reporting that the pro-oxidant activity of VC involving the reduction of Fe^3+^ to Fe^2+^ and subsequent formation of DHA was impeded by the iron chelator deferiprone [84]. Taken together, these findings suggest that the cytotoxic effects of plain VC are primarily associated with ROS production during its oxidation process via the iron-dependent Fenton reaction [22]. Also, our results underscore the importance of using VC that is kept under minimally oxidizing conditions to ensure maximum anti-tumor effects in the clinical treatment of cancer, including OS.

Notably, our OS results also align well with recent studies suggesting that differences in redox-active iron metabolism govern the differential susceptibility of cancer versus normal cells to pharmacological ascorbate in various preclinical cancer models, such as glioblastoma (GBM) [25], multiple myeloma [26,29], fibrosarcoma [27], liposarcoma [27], pancreatic cancer [28], and colorectal cancer [30]. Additionally, human clinical trial studies have demonstrated that magnetic resonance imaging (MRI) using T2* to detect changes in iron levels [[85], [86], [87]] and plasma biomarkers of iron [88] correlate with positive responses in humans treated with pharmacological VC, radiation, and chemotherapy. More recent studies have revealed that supplementation with FDA-approved, clinically available iron oxide nanoparticles, such as Ferumoxytol (FMX), an FDA-approved magnetite (Fe_3_O_4_) nanoparticle [89], and iron sucrose [90], can heighten the sensitivity of cancer cells to VC toxicity and enhance radio-chemo-sensitizing effects in GBM or colon cancer models [89]. These findings yield crucial mechanistic insights into the amplified anti-tumor efficacy of pharmacological VC mediated by intracellular iron in combination with other cancer inhibitors or radiation therapy.

Ferroptosis is well-known as an iron-dependent, non-apoptotic type of cell death resulting from ROS-inducing agents [68]. However, in contrast to treatment with the iron chelator DFO, we observed that treatment of OS cells with canonical ferroptosis inhibitors (Fer-1, Lip-1) had little to no effect on VC-induced cell death (Fig. 3H). These data correspond with the results of recent studies demonstrating the ineffectiveness of specific ferroptosis inhibitors in preventing VC-induced cell death, while DFO had complete inhibitory effects on HT-1080 fibrosarcoma, MCF-7 breast adenocarcinoma, and other pancreatic cancer cells [69,91]. Interestingly, conventional inhibitors of apoptosis and/or necrosis were unable to prevent VC-induced cell death. Correspondingly, recent studies have also reported the ineffectiveness of apoptosis and/or necrosis inhibitors in blocking VC-induced cell death in other types of cancer cells [34,49]. These observations suggest the involvement of alternative pathways that extend beyond the classical modes of cell death in response to high-dose VC treatment. In recent years, several non-apoptotic cell death pathways, such as parthanatosis and pyroptosis, have been proposed to mediate cell death induced by cytotoxic or genotoxic agents [72]. Given this, VC-induced cytotoxicity may involve multiple cell death pathways that act synergistically rather than relying on a single pathway.

While the extracellular ROS generated by VC oxidation is believed to be responsible for inducing cell death, the downstream signaling pathways that are activated by such ROS are not fully understood. In this regard, a recent study using pancreatic cancer cells has suggested that ROS-induced cytotoxicity upon treatment with pharmacological ascorbate may be partially mediated by dual oxidases 1 and 2 (DUOX1, DUOX2) of the NADPH oxidase (NOX) family of enzymes that not only produce H_2_O_2_ but can also be activated by H_2_O_2_ [56,92]. This implies the involvement of additional ROS-associated intracellular mechanisms in VC-induced cytotoxicity. Mitochondria, known as the primary producers of ROS in most cell types, play a crucial role in cellular energy production through OXPHOS [57,59]. Specifically, mitochondrial Ca^2+^ uptake through VDAC can stimulate OXPHOS, leading to ROS production from respiratory complexes I and III [74]. In addition, redox modifications regulate Ca^2+^ channels on the cell surface and intracellular organelles which, in turn, affect Ca^2+^ signaling that can influence the cellular generation of ROS from sources such as NADPH oxidases and mitochondria [93]. Notably, it has been reported that ERO1-α mediates stimulation of IP3R activity for Ca^2+^ release from ER during ER stress, thus triggering apoptosis [75]. In line with this, our findings, obtained through pharmacological and genetic perturbation analyses, suggest that mitochondrial Ca^2+^ overload and ROS production following Ca^2+^ release from the ER via an ERO1-α-IP3R pathway are crucial for VC-induced cell death (Fig. 4, Supplementary Fig. 6, Supplementary Fig. 7, Supplementary Fig. 8, Supplementary Fig. 9, Supplementary Fig. 10). Considering the extracellular and intracellular sources of ROS, it is speculated that Ca^2+^ release from the ER triggered by exogenous ROS via VC oxidation may amplify further ROS production by activating both NADPH oxidase enzymes and mitochondrial metabolic pathways, creating a vicious cycle of ROS production, ultimately contributing to VC-induced cell death.

Based on the Warburg hypothesis, which describes the preference of cancer cells for glycolysis over oxidative phosphorylation for energy production, ATP depletion and cell death in response to VC are mainly attributed to reduced glycolysis via inhibition of GAPDH activity by VC-induced H_2_O_2_ [17,18,20,55,94] (Fig. 8E). However, our global gene expression profiling data suggest that high-dose VC may impact ATP synthesis by down-regulating genes (Complex IV, V) involved in mitochondrial biogenesis and oxidative phosphorylation, while leaving nuclear glycolysis genes unaffected (Fig. 5, Fig. 6, Supplementary Fig. 12). Correspondingly, high-dose VC reduced MMP and ATP production and pretreatment with exogenous ATP nearly prevented VC-induced ROS and cytotoxicity (Fig. 7). These results provide evidence that mitochondrial genomic instability and respiratory dysfunction are important for instigating ATP depletion in VC-induced cytotoxicity. In support of this notion, a recent study using a pancreatic cancer cell model defective in mitochondrial respiration indicated that cancer cells that are able to use oxidative phosphorylation for ATP production are more susceptible to pharmacological 10.13039/100026939VC treatment, exhibiting increased 10.13039/100026054DNA damage and NAD + consumption as well as reduced ATP production compared to glycolysis-dependent cells [95]. Based on these findings, we propose a model in which ER-mitochondrial and glycolysis pathways interact synergistically to induce an energy crisis and cell death in response to high-dose VC (Fig. 8E).

Notably, an increasing number of studies suggest that most cancer treatments can lead to metabolic adaptation in cancer stem cells (CSCs), a subset of self-renewing cells within a tumor, which can confer therapy resistance by suppressing glycolysis and switching to mitochondrial OXPHOS [96,97]. Furthermore, combination therapy involving OXPHOS inhibition alongside oncogene-targeted therapy has been shown to selectively eradicate leukemia stem cells (LSCs) and pancreatic CSCs *in vitro* and *in vivo* [98,99]. Thus, our study provides a rationale for why independent or co-treatment with VC could be beneficial for therapy-resistant cancer including osteosarcoma and inhibition of tumor relapse by targeting mitochondrial metabolism. Further research in this area has promising implications for treating challenging, chemo-resistant osteosarcomas and other refractory cancers.

## 4 Methods and materials

### 4.1 Cell culture and reagents

U–2OS, 143B, Saos-2, and MNNG/HOS cells were obtained from the American Type Culture Collection (ATCC) and cultured in Dulbecco's Modified Eagle Medium (Gibco, Life Technologies, NY, USA) supplemented with 10 % fetal bovine serum (Sigma-Aldrich, MO, USA) and 1 % penicillin-streptomycin (Gibco, Life Technologies, NY, USA) at 37 °C with 5 % CO2. For transfections, siRNAs were introduced using Lipofectamine RNAiMAX (13778075, Thermo Fisher Scientific, Inc, Waltham, MA, USA), and DNA plasmids were transfected using Lipofectamine 3000 (L3000001, Thermo Fisher Scientific). Cells were fixed and stained using paraformaldehyde (P6148, Sigma-Aldrich), methanol (A412, Thermo Fisher Scientific), and crystal violet (405831000, Thermo Fisher Scientific).

### 4.2 Pharmacologic agents

Stock solutions (100 mM) of ascorbic acid (ASC, Sigma®), dehydroascorbic acid (DHA, 490-83-5, Cayman Chemical, Ann Arbor, MI, USA), l-ascorbic acid 2-phosphate (AA2P, A8960, Sigma) reduced glutathione (GSH, 70-18-8), and adenosine 5′-triphosphate (ATP, 987-65-5, Cayman Chemical) were prepared in complete culture medium or PBS and the pH was adjusted to neutral with sodium hydroxide. For extracellular calcium depletion, EGTA Buffer (0.5M, pH 8.0) was purchased (50-255-956, Fisher Scientific). Deferoxamine (DFO, Cayman Chemical), deferasirox (16753, Cayman Chemical), 2,2′-Bipyridyl (D216305, Sigma), penicillamine (23955, Cayman Chemical), 2-Aminoethyl diphenylborinate (2-APB, 64970, Cayman Chemical), dantrolene (14326, Cayman Chemical), DIDS (16125, Cayman Chemical), ferrostatin-1 (17729, Cayman Chemical), liproxstatin-1 (17737, Cayman Chemical), Z-VAD-FMK (A1902, Apexbio Technology LLC, Boston, MA, USA), necrostatin-1 (11658, Apexbio Technology LLC), IM-54 (13323, Cayman Chemical), erastin (17754, Cayman Chemical), BAPTA-AM (A1076, Sigma), Bobcat339 (408006, MedKoo Biosciences, Inc., Morrisville, NC, USA), DMOG (71210, Cayman Chemical), Disulfiram (15303, Cayman Chemical), BAY-876 (19961, Cayman Chemical), and STF-31 (11173, Cayman Chemical) stock solutions were prepared in dimethyl sulfoxide (DMSO, Sigma).

### 4.3 Clonogenic cell survival assay

Clonogenic cell survival assay was conducted following a protocol adapted from a recent study [75]. Briefly, Osteosarcoma cell lines (U2-OS, 143B, Saos-2, MNNG/HOS, and hFOB 1.19) were seeded at a density of 5 × 10^4^ cells/well in a 24-well plate (FB012929, Thermo Fisher Scientific). After 24 h, cells were treated with vehicle, VC, AA2P, and DHA for 3 h, or VC 5 mM for 3 h following a 30-min pre-treatment with drugs or transfected with siRNA for 48 h as indicated in the figures and treated with vehicle or VC 5 mM for 3 h. Cells were then trypsinized with 0.25 % trypsin-EDTA, and trypsin was inactivated by re-combining the cells with the media from the same treatment dish. Samples were centrifuged, re-suspended in fresh media, and counted using the Countess 3 FL automated cell counter (AMQAF2000, Thermo Fisher Scientific). A total of 400 cells were seeded into individual wells of a 24-well plate. Colonies were grown for 12–14 days in DMEM supplemented with 10 % FBS. The colonies were fixed with 4 % paraformaldehyde and stained with 0.5 % crystal violet solution in 25 % methanol.

### 4.4 3D tumor spheroid cultures

Osteosarcoma cells (U–2OS, 143B, Saos-2, and MNNG/HOS) were seeded (400 cells/well) onto low-attachment 96-well plates (#3474, Corning, NY, USA) in a 2D culture configuration. The growth medium employed was DMEM supplemented with 10 % fetal bovine serum (FBS). To minimize cell aggregation, a 1 % methylcellulose solution was introduced to achieve a final concentration of 0.5 % within the wells, thereby augmenting the viscosity of the medium. Over a period of one week, these cells were grown in a humidified incubator. Following this cultivation phase, the cells were treated with either vehicle or vitamin C. Subsequently, live-cell imaging of the developed tumor spheroids was conducted using a ZOE Fluorescent Cell Imager (#1450031, BioRad, CA, USA). For another set of experiments, 143B cells were seeded at a density of 5 × 10^4^ cells/well in a 24-well plate (FB012929, Thermo Fisher Scientific). After 24 h, cells were pre-treated with 10 μM BAPTA AM for 30 min, followed by treatment with vehicle or 5 mM VC for 3 h. Cells were trypsinized with 0.25 % trypsin-EDTA, and trypsin was neutralized by re-combining the cells with the media from the same treatment dish. Samples were centrifuged, re-suspended in fresh media, and counted using the Countess 3 FL automated cell counter. A total of 10,000 cells were seeded into individual wells of a low attachment 6-well plate (703011, Nest, Wuxi, Jiangsu, China) in a 2D culture configuration using DMEM supplemented with 10 % FBS. To minimize cell aggregation, 1 % methylcellulose solution was introduced to achieve a final concentration of 0.5 %. Cells were incubated for one week, and tumor spheroids were imaged using the ZOE Fluorescent Cell Imager.

### 4.5 Fluorescence labeling and live-cell imaging analysis

Fluorescence labeling and live-cell imaging analysis were conducted using the following methods. For assessing mitochondrial membrane potential in live cells, the TMRE Mitochondrial Membrane Potential Assay Kit (701310, Cayman Chemical) was utilized, following the manufacturer's protocol. To detect ROS in mitochondria, MitoROS™ 580 (16052, AAT Bioquest) was employed, according to the manufacturer's instructions. Apoptosis and necrosis were detected using the Annexin V-Cy3 Apoptosis Staining/Detection Kit with SYTOX (ab14144, Abcam) through a one-step staining procedure, following the manufacturer's protocol. Cells were seeded at a density of 2.5 x 10^4^ cells/well on a 96-well tissue culture-treated plate in complete culture medium. After 24 h of incubation, the cells were treated with either vehicle or vitamin C for a duration of 8–24 h. Subsequently, the complete culture medium was removed, and the cells were loaded with the fluorescent dyes during a 30-min incubation period. Visualization or measurement of the fluorescently-labeled cells was carried out with a BioTek Cytation 5 Cell Imaging Multimode Reader (Agilent Technologies, Inc., Santa Clara, CA, USA) with FITC and rhodamine filters, or by flow cytometry using the FL1 channel (Ex. 488 nm/Em 530 nm) for the SYTOX green dye and the FL2 channel for TMRE, MitoROS, and Annexin V-Cy3 (Ex. 543 nm/Em 570 nm).

### 4.6 Generation of HyPer red stable lines

U–2OS cells were transfected with the HyPer Red plasmid (pC1-HyPer-Red, 48249, Addgene) using Lipofectamine 3000 (L3000001, Thermo Fisher Scientific), following the manufacturer's protocol. After 48 h, the transfected U–2OS cells were subjected to selection in standard culture medium containing 0.8 mg/ml G418 (Invitrogen, Carlsbad, CA) for 10 days. Subsequently, single cells expressing a positive signal were sorted by FACS. For all live cell imaging experiments, HyPer Red cells were seeded at a density of 2.5 x 10^4^ cells/well on a 96-well tissue culture-treated plate in complete culture medium. Following a 24-h incubation period, cells were treated with either vehicle, VC alone, or the indicated drugs for 24 h, using the BioTek Cytation 5 Cell Imaging Multimode Reader.

### 4.7 Generation of bioluminescent cell lines

Bioluminescent cell lines expressing *pPer2-dLuc* were generated using the *pPer2-Luc* lentiviral reporter provided by A. C. Liu from the Department of Physiology and Functional Genomics, University of Florida. The 143B bioluminescent reporter lines were generated through lentivirus-mediated gene delivery following a stable transduction protocol, as previously described [100].

### 4.8 Cell viability assay

A total of 2.5 x 10^4^ U–2OS or 143B cells were seeded per well in 96-well plates. The cells were treated for 24 h with vehicle, VC, AA2P, or DHA, or with VC after a 30–60 min pretreatment with the indicated drugs, or the cells were transfected with siRNAs and treated 48 h post-transfection with vehicle or 5 mM VC, as indicated in the figures. Cell viability was then determined colorimetrically with Alamar Blue reagent (DAL1025, Thermo Fisher Scientific). Briefly, the media was removed from the plates by aspiration and a mixture of 100 μl of fresh media and 10 μl of Alamar Blue reagent was added to each well. The plates were incubated for 6 h at 37 °C, and fluorescence was monitored using 555 nm excitation and 595 nm emission on a Cytation 5.

### 4.9 Metabolic flux analysis

A total of 3 x 10^4^ cells (U–2OS or 143B) were seeded on Seahorse XF cell culture microplates (#103799, Agilent Technologies) and treated with varying concentrations (500 μM–10 mM) of vitamin C for 6 h or 24 h. The Seahorse XF analyzer (Agilent Technologies) was employed to conduct the assay, utilizing plates and media provided by Agilent for optimal performance. To execute this assay, specific reagents and media were utilized, including XF calibrant media (#100840, Agilent Technologies), XF DMEM media (#103575, Agilent Technologies), as well as cell culture microplates and flux packs (#103799, Agilent Technologies). Key compounds involved in the assessment, such as FCCP (#15218, Cayman Chemicals), oligomycin (#11342, Cayman Chemicals), rotenone (#13995, Cayman Chemicals), and antimycin A (#A8674, Sigma-Aldrich, St. Louis, MO, USA), were employed in accordance with established protocols. Following the administration of vitamin C, the Seahorse analyzer was calibrated following the manufacturer's instructions. The sensor cartridge was appropriately hydrated and degassed before commencing the assay. An hour preceding the assay, the cell media was substituted with XF DMEM, and cells were maintained at 37 °C in a non-CO2 incubator for degassing, aligning with the manufacturer's guidelines. Working solutions of FCCP, oligomycin, and antimycin A/rotenone were prepared in XF DMEM to the desired concentrations, adhering to specific port placements, as specified by the manufacturer's instructions. Upon completion of these preparatory steps, the cell culture microplate was positioned within the analyzer, and the designated assay protocol was established. Real-time measurements of Oxygen Consumption Rate (OCR) and Extracellular Acidification Rate (ECAR) were then conducted by the analyzer, enabling comprehensive assessment and analysis of metabolic parameters in response to the applied vitamin C treatments.

### 4.10 Measurements of ATP

A total of 3 x 10^4^ cells (U–2OS or 143B) were seeded in a 96-well plate and treated for 6 h with vitamin C, with final concentrations ranging from 250 μM to 20 mM. ATP concentrations were determined using the ATP Detection Assay Kit (700410, Cayman Chemical), according to the manufacturer's instructions.

### 4.11 Mitochondrial membrane potential (ΔΨm) measurements

A total of 3 x 10^4^ cells (U–2OS or 143B) were seeded in a 96-well plate and treated with 5 mM vitamin C for 2, 4 and 6 h ΔΨm determinations were carried out using the TMRE Mitochondrial Membrane Potential Assay Kit (ab113852, Abcam).

### 4.12 Isolation and quantitation of protein samples

Cells were treated and prepared, as described above, and were lysed in RIPA protein isolation buffer (150 mM NaCl, 1 % NP-40, 50 mM Tris, pH 8.0) supplemented with 1 % protease inhibitor cocktail (Sigma), 1 % phosphatase inhibitor cocktail (Sigma®), and 1 mM PMSF. Samples were incubated on ice for 30 min and centrifuged at 14000×*g* for 15 min at 4 °C. The supernatant was used for protein analysis and stored at −80 °C. Protein samples were quantified using the Pierce™ BCA Protein Assay Kit (Thermo Scientific) according to the manufacturer's guidelines.

### 4.13 Immunoblotting and antibodies

U–2OS cells were treated and prepared, as described above, and were lysed in cell lysis buffer (9803S, Cell Signaling) composed of 20 mM Tris-HCl, pH 7.5, 150 mM NaCl, 1 mM Na_2_EDTA, 1 mM EGTA, 1 % Triton X-100, 2.5 mM sodium pyrophosphate, 1 mM beta-glycerophosphate, 1 mM Na_3_VO_4_, and 1 μg/ml leupeptin, supplemented with 1 mM PMSF (Sigma), 1 % phosphatase inhibitor cocktail (Sigma), and 1 mM PMSF protease inhibitor (36978, Thermo Fisher, Inc). Samples were incubated on ice for 30 min and centrifuged at 14000×*g* for 15 min at 4 °C. The supernatant was used for protein analysis and stored at −80 °C. Protein samples were quantified using the Pierce™ BCA Protein Assay Kit (23227, Thermo Scientific, Inc.), according to the manufacturer's guidelines. SDS-PAGE was performed using 4–20 % Criterion™ TGX™ Precast Midi Protein Gels (5671094, Bio-Rad). Proteins were transferred onto Millipore 0.45 μM PVDF membranes. Immunoblotting was performed using TBS-Tween (0.1 %) containing 5 % non-fat dry milk for blocking the membrane and for antibody solutions. For each experiment, at least *three* independent measurements were carried out. The following antibodies were used: anti-MT-ATP6 (14020, Cell Signaling, Danvers, MA, USA), anti-MT-CO1 (55159S, Cell Signaling), anti–caspase-3 (9662S, Cell Signaling), anti-cleaved caspase-3 (9661S, Cell Signaling). Anti-GAPDH (2118, Cell Signaling), anti-tubulin (ab18251, Abcam), and anti-β-actin (4967S, Cell Signaling) antibodies were used to detect loading controls.

### 4.14 Transfection of siRNA

Small interfering RNAs (siRNAs) targeting human *ITPR1* were generated with the following synthesized primers: forward - 5′-AGACAGAAAACAGGAAAUUTT-3′ and reverse - 5′-AAUUUCCUGUUUUCUGUCUCA-3′ (GeneLink, Inc. Orlando, FL, USA) [101]. siRNAs targeting human *ITPR2* (SI00034552) and *ITPR3* (SI00034587) were purchased from Qiagen. Human ERO1a small interfering RNA (siRNA) was synthesized with the following sequences: forward primer, 5′-ACCAGACAAGAAAUAGUAUCAUU-3′; and reverse primer, 5′-AAUGAUACUAUUUCUUGUCUGGU-3′ (GeneLink, Inc). Transfection of U–2OS cells with 100 pmol of siRNA per well was conducted with Lipofectamine RNAiMAX Transfection Reagent (13778075, Thermo Fisher Scientific, Inc.) according to the manufacturer's instructions.

### 4.15 RNA extraction, reverse transcription, and quantitative PCR

Total RNA was isolated from cells (U–2OS) using the RNeasy Plus Mini Kit (74134, Qiagen), according to the manufacturer's protocol. Equal amounts of complementary DNA were synthesized using the Invitrogen Superscript II RT First-Strand Synthesis System, according to the manufacturer's protocol, using 2 μl of random hexamers and 9 μl of RNA (Life Technologies, Carlsbad, CA, USA). qPCR assays were performed using the SYBR Green PCR Master Mix (1708880, BioRad, Hercules, CA, USA) and 10 μM of the forward and reverse primers (Supplementary Table S1). All qPCR reactions were conducted at 50 °C for 2 min, 95 °C for 10 min, and then 40 cycles of 95 °C for 15 s and 60 °C for 1 min. The specificity of the reaction was assessed by melting curve analysis. The relative gene expression of each sample was quantified using the comparative Ct method. Samples were normalized to GAPDH, which was used as an endogenous control for all experiments. Experiments were performed using a ViiA7 Real-Time PCR machine (Thermo Fisher Scientific).

### 4.16 RNA-sequencing

Total RNA was isolated from U–2OS cells using Trizol reagent (15596018, Invitrogen), following the manufacturer's instructions. The RNA library preparation and transcriptome sequencing were performed by Novogene Co., LTD (Davis, CA, USA). Genes with an adjusted *p*-value <0.05 and |log2(FoldChange)| > 0 were considered differentially expressed for GO and KEGG Pathway Enrichment Analysis.

### 4.17 *In vivo* xenograft experiments

Male, 6∼8-week-old BALB/c nude immunodeficient mice (CAnN.Cg-Foxn1nu/Crl, Charles River) were used for orthotopic transplantation of osteosarcoma cells. The mice were anesthetized by exposing them to 2–3% isoflurane in oxygen. A small amount of ophthalmic ointment was applied to the eyes to prevent dryness while under anesthesia. Each mouse was placed in a supine position. The ankle of the mouse was held using the thumb and index finger, and the injection site on the tibia was disinfected with a 70 % ethanol swab. To expose the proximal tibia plateau, the ankle joint was rotated outward, moving the tibia and fibula, and the knee joint was bent to an appropriate position. The needle, attached to a 1 mL syringe, was pointed towards the injection site, ensuring that it was parallel to the long axis of the tibia. Proper positioning of the needle within the medullary canal was confirmed by a prominent movement. A micro-volume syringe loaded with 143B-Luc osteosarcoma cell suspension was used to slowly inject approximately 10–20 μl of the cell suspension (approximately 1 x 10^6^ cells) into each mouse's tibia without applying excessive pressure. After removing the micro-volume syringe, the injection site was gently pressed with a cotton swab for 20–30 s. Mice were raised in specific pathogen-free conditions. All procedures were done in an aseptic cabinet with sterile tools. The mice were monitored for 3 consecutive days after the procedure for decreases in weight, mobility, physical appearance, and body condition. After this, they were monitored once weekly for the same changes. When detectable tumors grew after ∼4 weeks, the mice were injected intraperitoneally with vehicle (water) or a vitamin C (3.3 g/kg) solution on a daily basis. Tumor volumes (mm^3^) were measured weekly for three weeks using a digital caliper. Tumor growth was assessed using a digital caliper, and tumor weights were calculated using the formula: (length × width^2)/2. In all animal experiments, mice were sacrificed and tumors were harvested when the mean tumor diameter reached >20 mm. All animal procedures were approved by the Washington State University Animal Care and Use Committee and conformed to National Institutes of Health guidelines.

### 4.18 *In vivo* bioluminescence

Following vehicle/VC treatment in 143B-Luc tumor-bearing mice, as described above, *in vivo* bioluminescence imaging was conducted to assess luciferase reporter intensity in the xenografted tumors using the PerkinElmer IVIS Spectrum CT. Subsequent to intraperitoneal administration of 6.6 mg/kg CycLuc1 (AOB1117, Aobious Gloucester, MA, USA), mice were anesthetized with 2–3% isoflurane in oxygen. Ophthalmic ointment was applied topically to mitigate ocular dryness during anesthesia. Mice were then immobilized in a standardized position within the imager and subjected to imaging utilizing Living Image software.

### 4.19 Histology of human osteosarcoma xenograft tissues

The 143B-derived human osteosarcoma (OS) tumor tissues from vehicle and VC-treated mice were fixed in 4 % Paraformaldehyde (PFA, Sigma-Aldrich) for one day at 4 °C and subsequently stored in 70 % Ethanol at 4 °C. Samples underwent sequential immersion in 80 % ethanol, 95 % ethanol, and three successive rounds of 100 % ethanol, with each immersion lasting for 1 h at a controlled temperature of 50 °C. Subsequently, the samples were immersed in Xylene 1 (ES609, Azer Scientific, Morgantown, PA, USA) for 1 h, followed by immersion in Xylene 2 for an additional hour, both maintained at 55 °C. Following the xylene clearing steps, the samples were subjected to paraffin wax infiltration, with each immersion in paraffin wax lasting for 1 h, 40 min, and 30 min, consecutively, at a temperature of 60 °C. The processed tissue samples were then sliced into sections of 5 μm thickness using a microtome and transferred onto glass slides. Subsequently, the slides were allowed to dry overnight before further analysis.

### 4.20 Hematoxylin and eosin staining

Tissue sections mounted on slides were immersed in xylene for 2 min, repeated three times to ensure efficient deparaffinization. Subsequently, the sections were briefly immersed in 100 % ethanol for 1 min, followed by sequential immersions in 95 % ethanol, 70 % ethanol, and running water, each for 1 min, to facilitate hydration. The sections were then stained with 2X Hematoxylin (10143-146, VWR, Radnor, PA, USA) for 1 min and 45 s, followed by a 2-min rinse in running water. A brief 15-s immersion in acetic acid was conducted for followed by a 1-min rinse in running water. The sections were then subjected to a 1-min immersion in Scotts Tap Water substitute (26070-07, Electron microscopy sciences, Hatfield, PA, USA) followed by rinsing in running water for 1 min. Subsequently, the sections were dehydrated through sequential immersions in 95 % ethanol for 1 min, followed by immersion in Eosin Y for 1 min. Further dehydration was achieved through immersions in 95 % ethanol and three rounds of 100 % ethanol, each for 1 min. Finally, the sections were cleared in xylene for 1 min, repeated twice, before being mounted for microscopic examination using Zeiss One microscope.

### 4.21 Immunohistochemistry

Tissue sections mounted on slides were subjected to a dewaxing and rehydration process to prepare them for immunohistochemical staining. Slides were submerged successively in xylene 1 for 20 min, xylene 2 for 10 min, 100 % ethanol for 3 min, 90 % ethanol for 3 min, 70 % ethanol for 3 min, 60 % ethanol for 3 min, and 50 % ethanol for 3 min. Subsequently, the slides were immersed in 1X Citrate Buffer and subjected to microwave treatment for 14 min to facilitate antigen retrieval, followed by cooling at room temperature for 1 h. Afterward, the slides underwent two washes in 1X TBST (Tris-buffered saline with Tween) for 5 min each. To block endogenous peroxidase activity, the tissue sections were incubated with 3 % H_2_O_2_ at room temperature for 10 min, followed by another wash with 1X TBST. Blocking of non-specific binding sites was achieved by incubating the slides with IHC blocking buffer at room temperature. The primary antibody targeting ATP6 (55313-1-AP, Proteintech, Rosemont, IL, USA) was applied to the slides at a dilution of 1:100 in 1X TBS and incubated overnight at 4 °C. The next day, the slides were washed with 1X TBST to remove excess primary antibody. Subsequently, the secondary antibody, Goat anti-Rabbit IgG Secondary Antibody (VC003-025, Novus Biologicals, Centennial CO, USA), pre-diluted, was applied to the slides and incubated for 1 h at room temperature, followed by two washes with 1X TBST. Detection of antibody binding was achieved by adding DAB solution (8059, Cell Signaling Technology), followed by counterstaining with Harris Hematoxylin (3801562, Leica Biosystems, Deer Park IL, USA) for 3 min. After staining, the slides were dehydrated by successive immersion in ethanol solutions of increasing concentrations: 60 % ethanol for 3 min, 80 % ethanol, 95 % ethanol, and 100 % ethanol for 3 min each, followed by 100 % ethanol for an additional 3 min, and finally in xylene 1 for 5 min. The dehydrated slides were mounted and left to air-dry overnight in a fume hood. Subsequently, brightfield images were captured using a Zeiss One microscope.

### 4.22 Statistical analysis

Statistical calculations for all experiments were performed using GraphPad Software (version 10). Image J software was used for quantifying fluorescence intensities, immunoblots, and spheroid sizes. Statistical analysis of such quantitation was performed using GraphPad software. Significance was determined using two-way ANOVA, one-way ANOVA, and unpaired Student's t-tests, as appropriate. *P* values < 0.05 were interpreted as statistically significant.

## Funding

This work was supported by the Hennings Cancer gift in the Elson S. Floyd College of Medicine (GF003245 to Y. L.).

## Data availability

Data will be made available on request.

## CRediT authorship contribution statement

**Prajakta Vaishampayan:** Data curation, Formal analysis, Investigation, Methodology, Resources, Software, Validation, Visualization, Writing – review & editing. **Yool Lee:** Conceptualization, Data curation, Formal analysis, Funding acquisition, Investigation, Methodology, Project administration, Resources, Software, Supervision, Validation, Visualization, Writing – original draft, Writing – review & editing.

## Declaration of competing interest

Authors declare that they have no competing interests.
