# Supplementary material for: Redox-active vitamin C suppresses human osteosarcoma growth by triggering intracellular ROS-iron–calcium signaling crosstalk and mitochondrial dysfunction
Source: Redox Biol. 2024 Jul 26;75:103288. doi: 10.1016/j.redox.2024.103288 (PMC11342202; doi:10.1016/j.redox.2024.103288)
Supplement: Multimedia component 1 [file mmc1.docx]

**Supplementary Materials**

**Title:** **Redox-active Vitamin C Suppresses Human Osteosarcoma Growth by Triggering Intracellular ROS-Iron-Calcium Signaling Crosstalk and Mitochondrial Dysfunction**

**Authors:** Prajakta Vaishampayan^1^, Yool Lee^1,2,3,4^*,

*Corresponding author. Email: yool.lee@wsu.edu

^1^ Department of Translational Medicine and Physiology, Elson S. Floyd College of Medicine, Washington State University, Spokane, WA 99202, USA

^2^ Department of Integrative Physiology and Neuroscience, College of Veterinary Medicine, Washington State University, Pullman, WA 99164, USA

^3^ Sleep and Performance Research Center, Washington State University, Spokane, WA 99202, USA

^4^ Steve Gleason Institute for Neuroscience, Washington State University, Spokane, WA 99202, USA

**This file includes:**

**Supplementary Fig. 1.** Clonogenic cell survival assay for the dose-dependent effect of plain, reduced, and oxidized forms of vitamin C on colony formation in human OS cells

**Supplementary Fig. 2**. The dose-dependent cytotoxicity of VC is blocked by catalase antioxidant cotreatment.

**Supplementary Fig. 3**. Comparative analysis of the tumor spheroid formation efficiency of human OS cells under 3D culture conditions.

**Supplementary Fig. 4**. Vitamin C dose-dependently suppresses 3D-cultured OS spheroid growth.

**Supplementary Fig. 5.** Dose-and time-dependent effects of vitamin C, AA2P, and DHA on ROS generation in Hyper Red reporter U-2OS cells.

**Supplementary Fig. 6**. Vitamin C induces non-apoptotic cell death in human OS cells in a Ca^2+^-dependent manner.

**Supplementary Fig. 7**. Intracellular Ca^2+^ pathways are critical for VC-induced ROS production and OS cell death via non-canonical apoptotic and necrotic mechanisms.

**Supplementary Fig. 8**. High-dose vitamin C induces an increase in intracellular calcium levels.

**Supplementary Fig. 9**. Clonogenic cell survival analysis of the effect of various ion chelators and metabolic modulators on VC-induced cytotoxicity.

**Supplementary Fig. 10**. The effects of ITPR or ERO1A gene knockdown on high-dose VC-induced cytotoxicity in human OS cells.

**Supplementary Fig. 11**. Transcriptome analysis of U-2OS cells treated with and without VC

**Supplementary Fig. 12**. Comparative analysis of the effects of high-dose vitamin C on the expressions of genes in the glycolysis and mitochondrial pathways in U-2OS cells.

**Supplementary Fig. 13**. Dose-dependent effects of vitamin C on mitochondrial gene expression.

**Supplementary Fig. 14**. Metabolic flux and ATP analyses of 143B cells treated with varying doses of vitamin C.

**Supplementary Fig. 15**. Histological analysis of the effects of high-dose vitamin C on human OS xenograft tumors in mice.

**Supplementary Table 1**. qPCR primers for mitochondrial and ITPR genes.

**Other Supplementary Material for this manuscript includes the following:**

Supplementary data file 1 (Microsoft Excel format). Numerical data for RNA Seq. data analysis

**Supplementary Fig. 1**

**
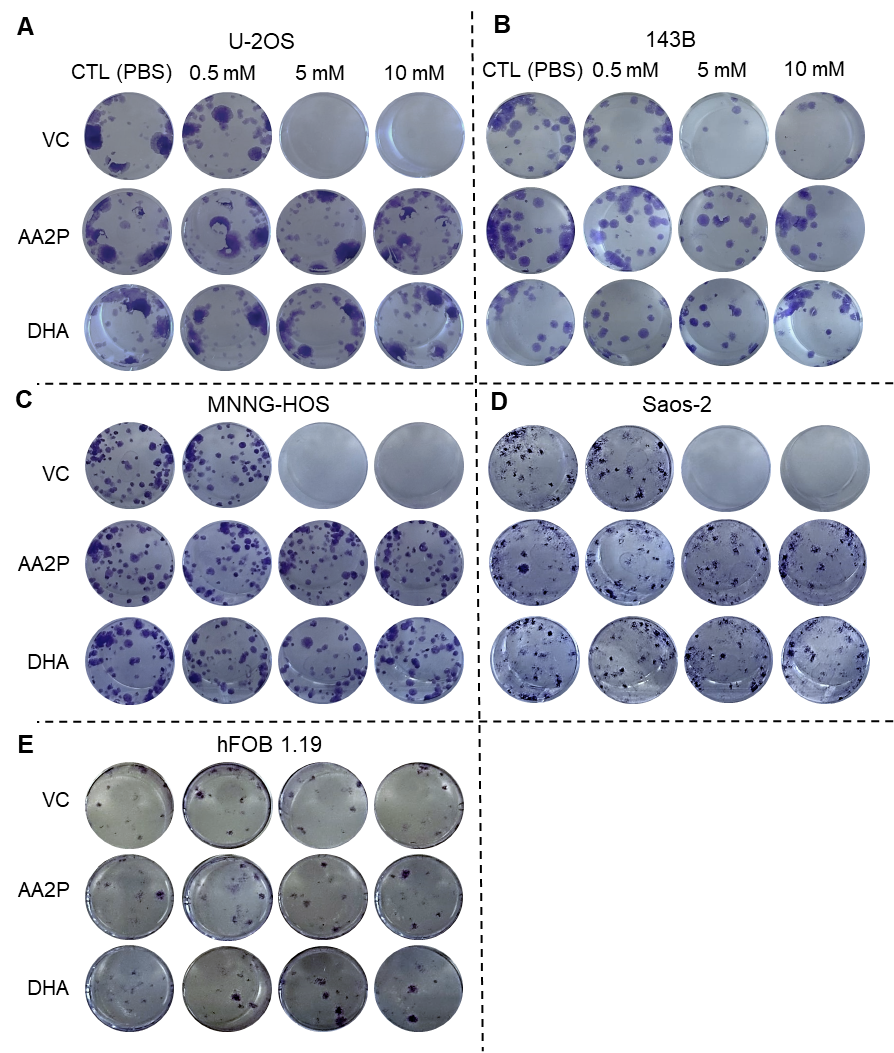
**

**Supplementary Fig. 1.** **Clonogenic cell survival assay for the dose-dependent effect of plain, reduced, and oxidized forms of vitamin C on colony formation in human OS cells**. (A) U-2OS, (B) 143B, (C) MNNG-HOS, (D) Saos-2, and (E) hFOB 1.19 cells were pre-treated with vitamin C (VC), ascorbic acid 2-phosphate (AA2P), and dehydroascorbic acid (DHA) at the indicated concentrations (0.5 ~ 10 mM). After 3 hours, cells were trypsinized and seeded at a density of 200 cells/well for a clonogenic cell survival assay. After 10 days of incubation, cells were fixed with 4% PFA and stained with 0.5% crystal violet before imaging. Representative images are shown from three independent experiments.

**Supplementary Fig. 2**

**
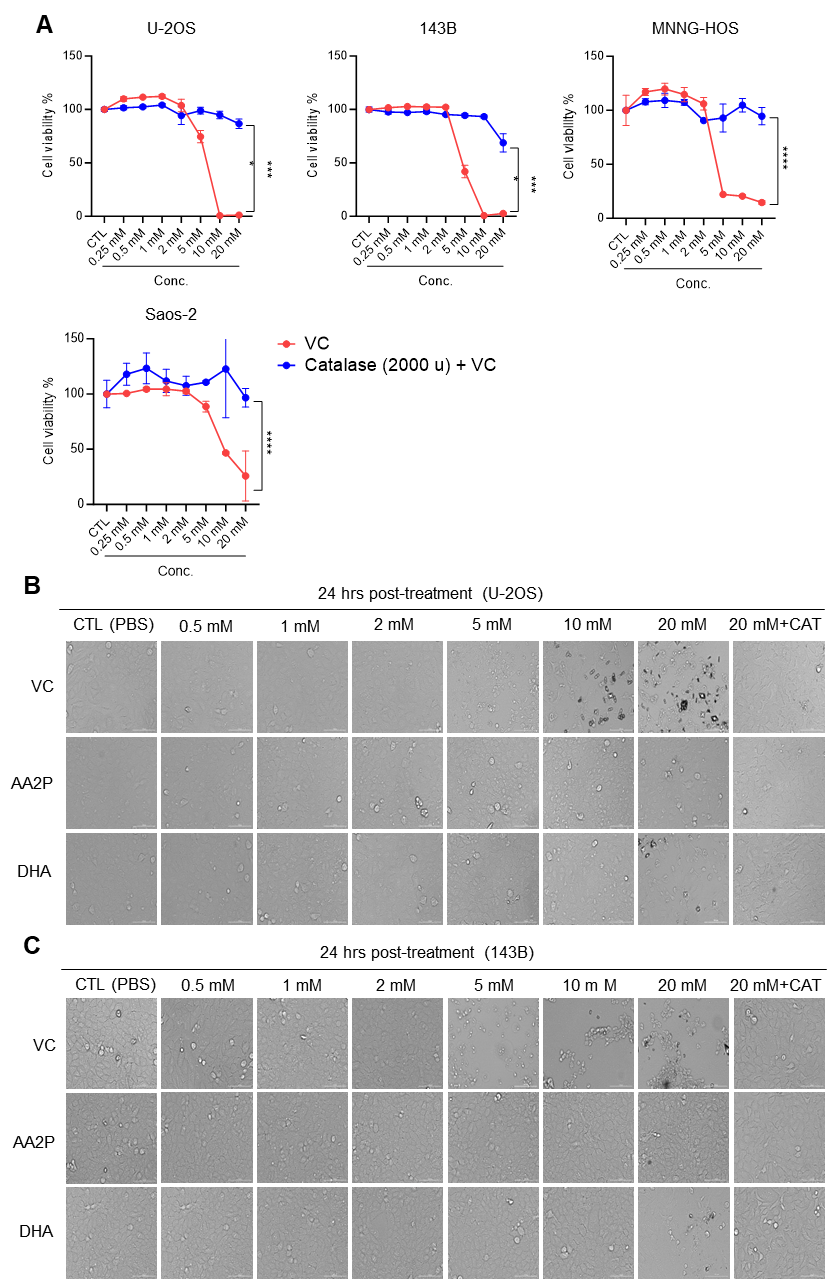
**

**Supplementary Fig. 2.** **The dose-dependent cytotoxicity of VC is blocked by catalase antioxidant cotreatment**. (**A**) Alama blue cell viability analysis of U-2OS, 143B, MNNG-HOS, and Saos-2 cells after 24 hours of treatment with different doses (0.25 ~ 20 mM) of VC (Red) with or without treatment with catalase (CAT, 2000u, blue), as indicated. (**B, C**) Representative brightfield images of the dose-dependent effects of VC, AA2P, and DHA on cell viability in U-2OS (B) and 143B (C) cells in the presence or absence of catalase (CAT, 2000u), as indicated.

**Supplementary Fig. 3**


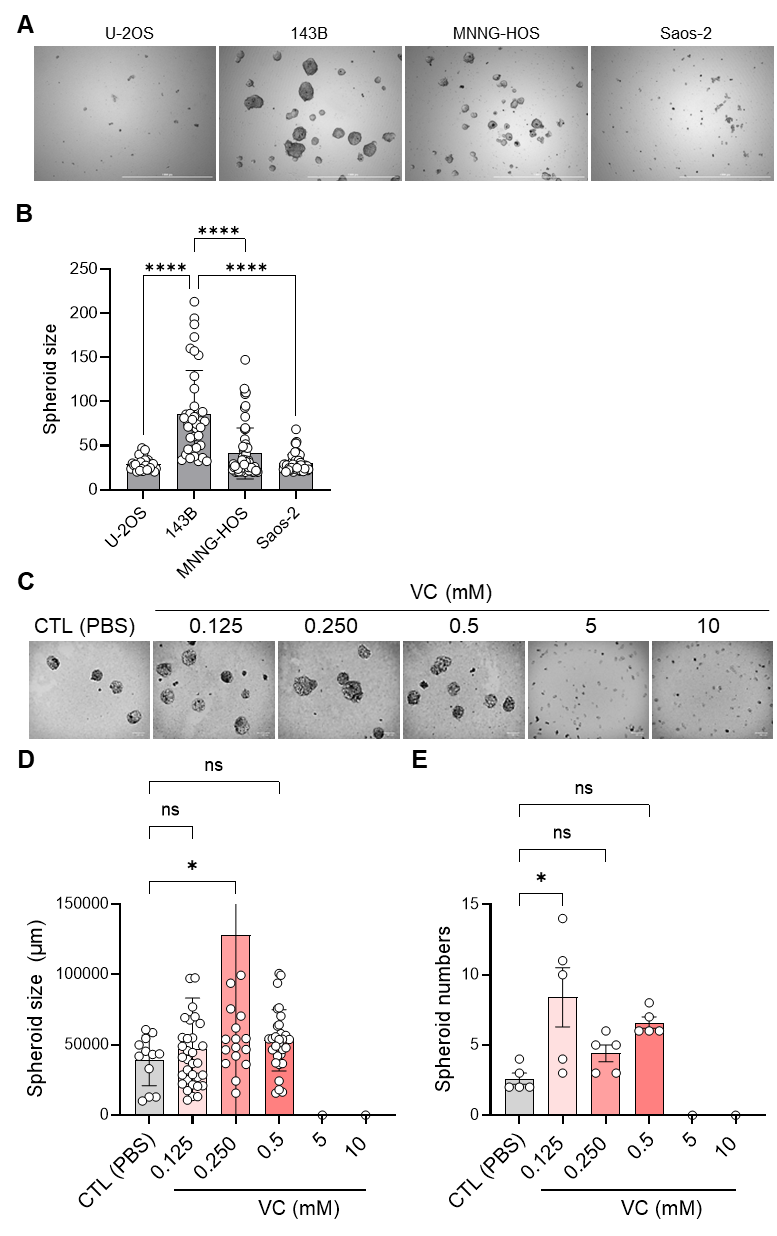


**Supplementary Fig. 3. Comparative analysis of the tumor spheroid formation efficiency of human OS cells under 3D culture conditions.** (**A**) Representative brightfield images of 3D-cultured spheroids derived from various human osteosarcoma (OS) cells, as indicated. To initiate spheroid formation, 4 x 10^2^ OS cells were cultured in 3D culture media in each well of a 96-well ultra-low attachment plate and incubated for one week. Images were captured upon the detection of visible spheroids. (**B**) Bar graph showing the sizes of the spheroids shown in A (*n* > 30 per cell type) determined using Gen 5 analysis software. *****p* <0.0001 by two-way ANOVA with Tukey's multiple comparisons test. (**C**) Dose-dependent effect of Vitamin C (VC) on spheroid forming capacity of 143B OS cells. 10,000 cells were plated under 1% methylcellulose-containing culture media on a 6-well low attachment plate and treated with PBS (CTL) or different concentrations of VC (0.125 ~ 10 mM) as indicated. After a week, representative brightfield images of spheroids were taken for each concentration of VC. (**D**) Quantitative analysis of spheroid sizes in (C) using ImageJ software. (**E**) Quantitative analysis of average spheroid numbers formed by each concentration for each field of view (n=5) that the pictures were taken. *p < 0.05, by one-way ANOVA with Tukey's multiple comparisons test; ns, non-significant.

**Supplementary Fig. 4**


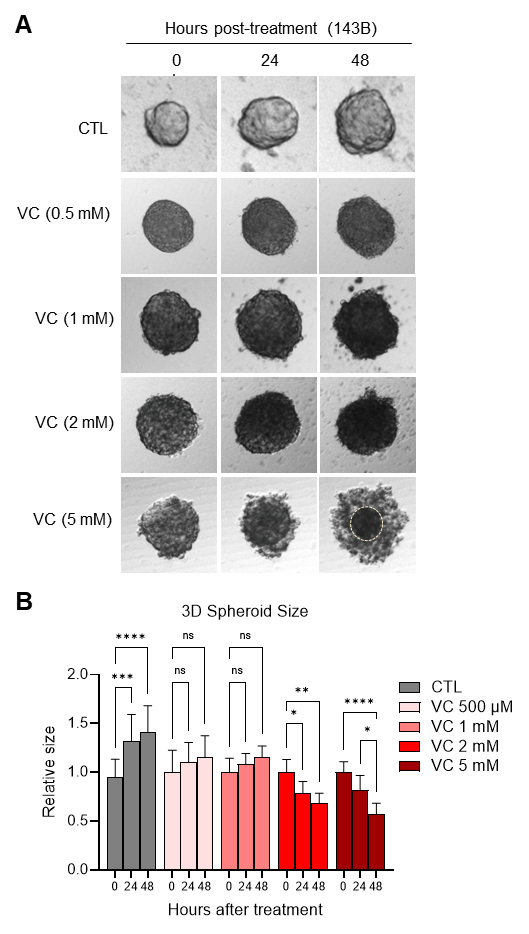


**Supplementary Fig. 4. Vitamin C dose-dependently suppresses 3D-cultured OS spheroid growth.** (**A**) Representative brightfield images showing the dose-dependent effects of vitamin C (VC) on OS spheroids. 4 x 10^2^ 143B cells were cultured in 3D culture media in each well of a 96-well ultra-low attachment plate and incubated for one week. Once detectable spheroids had formed, images were taken at the indicated times (0 ~ 48 hours) after treatment with vehicle (CTL) or VC at the indicated doses (0.5 ~ 5 mM). The dashed yellow circle indicates the residual spheroidal body of the inner core that remained after the disintegration of the outer cell debris due to VC treatment. (**B**) Bar graph representing the statistical analysis of the dose and time-dependent effects of VC on the growth rate of the 3D-cultured 143B spheroids shown in A. **p* < 0.05, ***p* < 0.005, ****p* = 0.0009, *****p* <0.0001 by two-way ANOVA with Tukey's multiple comparisons test; ns, non-significant. Data are representative of three independent experiments.

**Supplementary Fig. 5**

**
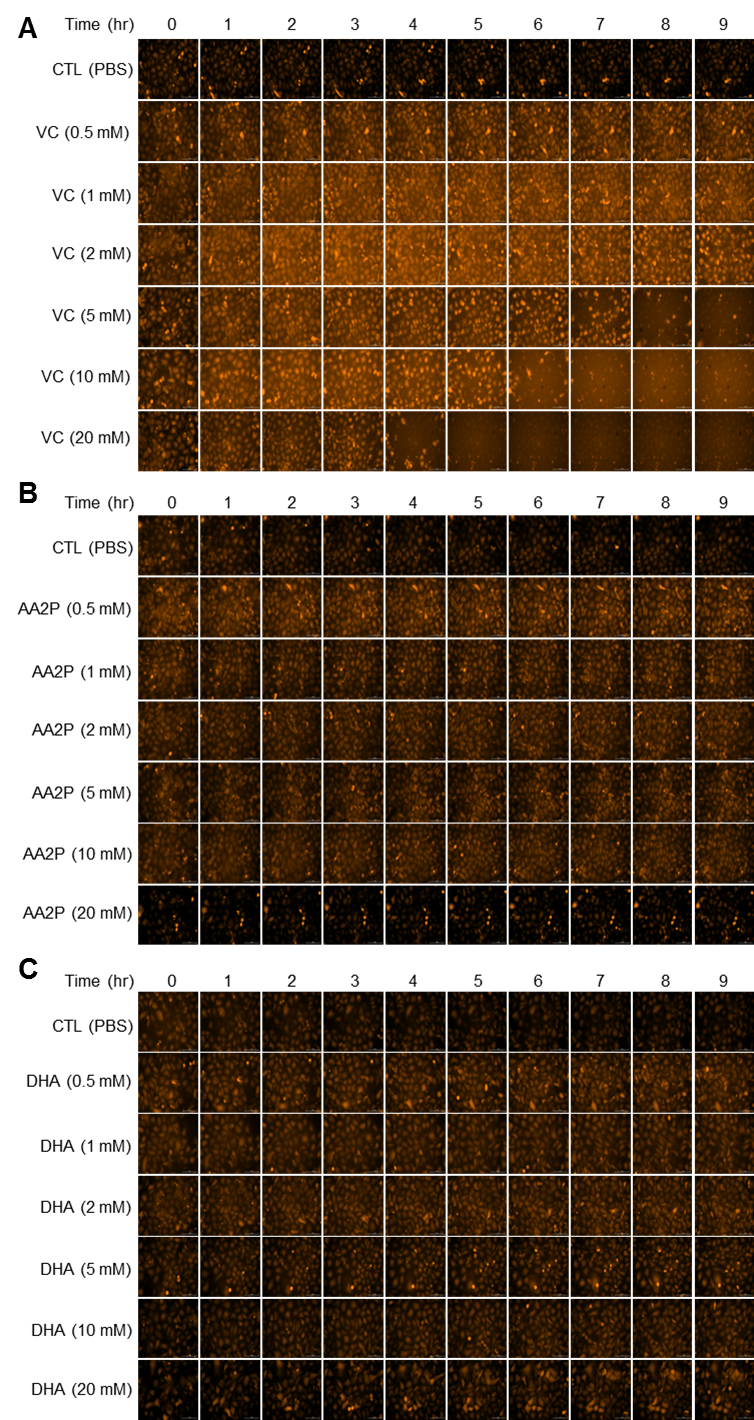
**

**Supplementary Fig. 5. Dose-and time-dependent effects of vitamin C, AA2P, and DHA on ROS generation in Hyper Red reporter U-2OS cells.** (**A–C**) Dose-and time-dependent effects of VC, AA2P, and DHA on intracellular H_2_O_2_ levels in U-2OS cells stably expressing HyPer Red (cpmApple) probe. The time-lapse live-cell images were visualized with a red fluorescence filter set (excitation [Ex] 575 nm/emission [Em] 605 nm).

**Supplementary Fig. 6**

**
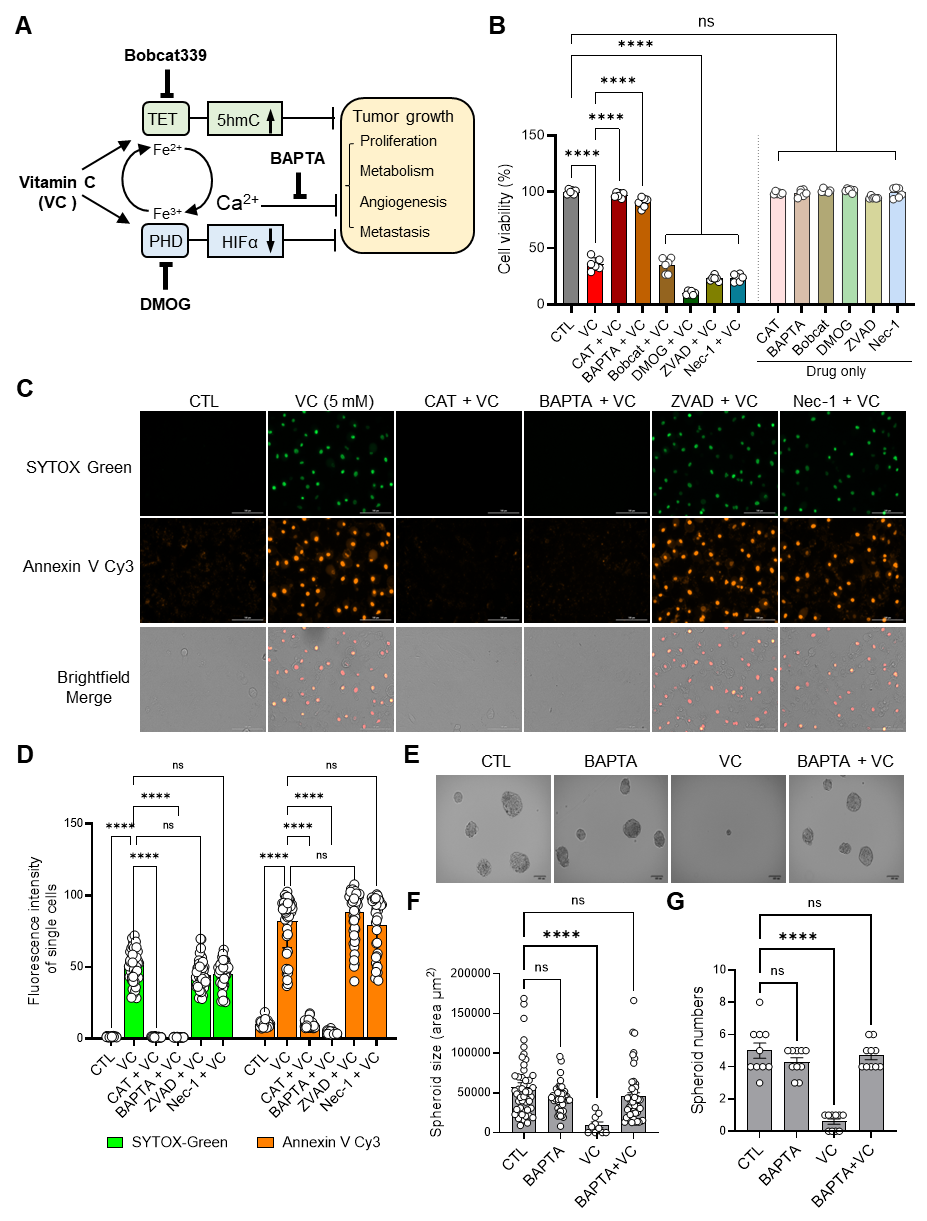
**

**Supplementary Fig. 6. Vitamin C induces non-apoptotic cell death in human OS cells in a Ca^2+^-dependent manner.** (**A**) Schematic diagram illustrating pharmacological inhibition of Fe^2+^- and 2-oxoglutarate-dependent dioxygenases (e.g., ten-eleven translocation enzymes [TET], prolyl-hydroxylase domain-containing proteins [PHD], and hypoxia-inducible factor [HIF] hydroxylases) as well as Ca^2+^ pathways. (**B**) Cell viability assays of U-2OS cells treated with

vitamin C (VC, 5 mM) and/or inhibitor (catalase [CAT, 2000u], BAPTA AM intracellular calcium inhibitor [BAPTA, 10 μM], Bobcat339 DNA Methyltransferase Inhibitor [Bobcat, 33 μM], Dimethyloxallyl Glycine [DMOG, 2 μM), Z-VAD-FMK Pan Caspase Inhibitor [ZVAD, 10 μM], Necrostatin-1 RIP1 inhibitor (Nec-1 [10 μM]) alone or in combination, as indicated, for 24 hours. The data show means normalized to untreated wells (Δ) ± SD of three independent experiments. (**C**) Representative images of cells loaded with SYTOX Green necrosis or Annexin V Cy3 apoptosis staining dyes following exposure to VC (5 mM) alone or in combination with the indicated drugs for 5 hours. Specific fluorescence filter sets were used for the detection of SYTOX Green (Ex. 488 nm/Em. 530 nm) and Annexin V-Cy3 (Ex. 543 nm/Em. 570 nm). (**D**) Quantitation of the intracellular SYTOX Green and Annexin V-Cy3 signals in individual cells (*n* = 30 ~ 50) treated with the indicated drugs (as shown in C using Image J. Original fluorescent images of cells were converted to white and black mode, and the signal intensities were quantified using Image J. *****p* <0.0001 by two-way ANOVA with Tukey's multiple comparisons test. (**E**) 143B cells were pretreated with PBS and BAPTA (10 µM) for 30 minutes before VC (5 mM) treatment alone or in combination as indicated for 6 hours. The cells were then trypsinized and seeded in a low attachment 6-well plate at a density of 10,000 cells/well under 1% methylcellulose condition. After 1 week, representative brightfield images were captured. (**F**, **G**) Spheroid size (F) and numbers (G) analysis were conducted using ImageJ and GraphPad Prism software. *p < 0.05, **p < 0.001, ****p <0.0001 by one-way ANOVA with Tukey's multiple comparisons test. ns, non-significant.

**Supplementary Fig. 7**

**
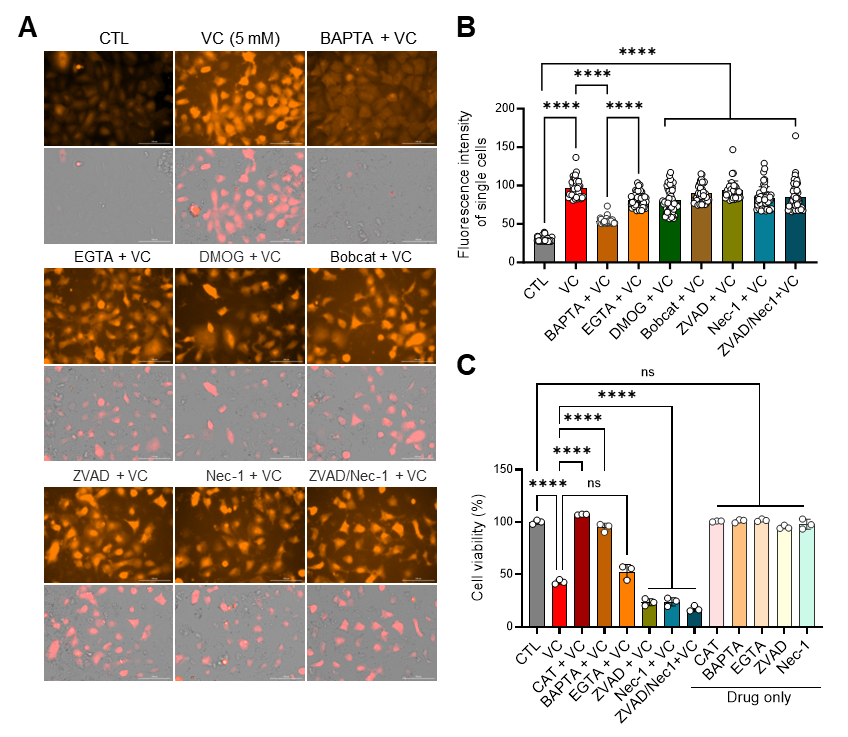
**

**Supplementary Fig. 7. Intracellular Ca^2+^ pathways are critical for VC-induced ROS production and OS cell death via non-canonical apoptotic and necrotic mechanisms.** (**A**) Representative images of HyPer Red U-2OS cells treated with VC (5 mM) alone or in combination with the indicated drugs (catalase [CAT, 2000u], BAPTA AM intracellular calcium inhibitor [BAPTA, 10 μM], EGTA extracellular calcium inhibitor (EGTA, 5 mM), Bobcat339 DNA methyltransferase inhibitor [Bobcat, 33 μM], Dimethyloxallyl Glycine [DMOG, 2 μM), Z-VAD-FMK pan caspase inhibitor [ZVAD, 10 μM], Necrostatin-1 RIP1 inhibitor (Nec-1 [10 μM]). Cells were pretreated with the indicated drugs for 30 min before treating with VC (5 mM) for 5 hours. Live-cell images were acquired using the red fluorescence filter cube (Ex. 531 nm/Em. 593 nm) in a Cytation 5 multi-mode reader. (**B**) Quantitation of the intracellular HyPer Red signal in individual cells (*n* = 40 ~ 60) treated with the indicated drugs, as shown in A. The intensity of the fluorescence was quantified using Image J. (**C**) Cell viability assays of U-2OS cells treated with VC (5 mM) alone or in combination with the indicated drugs for 24 hours. *****p* <0.0001 by two-way ANOVA with Tukey's multiple comparisons test. ns., non-significant. The data are shown as means normalized to untreated wells (Δ) ± standard deviation (SD) of experiments conducted in triplicate.

**Supplementary Fig. 8**


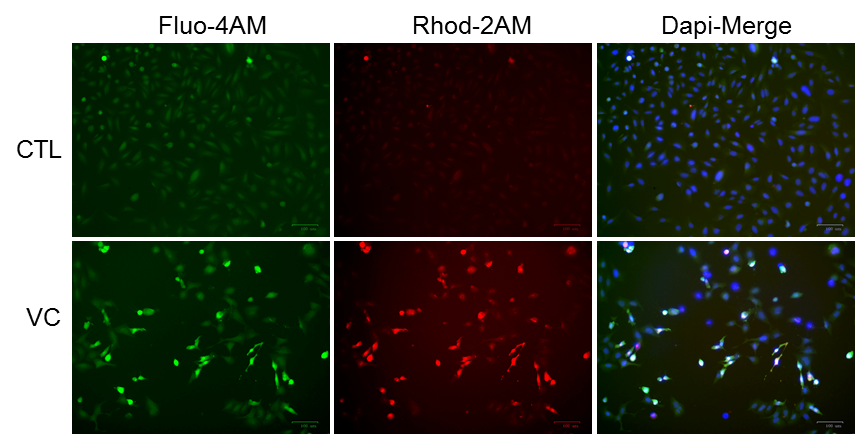


**Supplementary Fig. 8. High-dose vitamin C induces an increase in intracellular calcium levels.** Representative images of U-2OS cells loaded with Fluo-4AM intracellular calcium fluorescence indicator (Green, λex=494 nm, λem=516 nm) and Rhod-2AM mitochondrial calcium fluorescence indicator (Red, λex=557 nm, λem=581 nm) following exposure to VC (5 mM) for 5 hours. Specific fluorescence filter sets (Fluo-4AM green: Ex.488 nm/Em. 530 nm and Rhod-2AM red: Ex. 543 nm/Em. 570 nm) were used for dye detection.

**Supplementary Fig. 9**


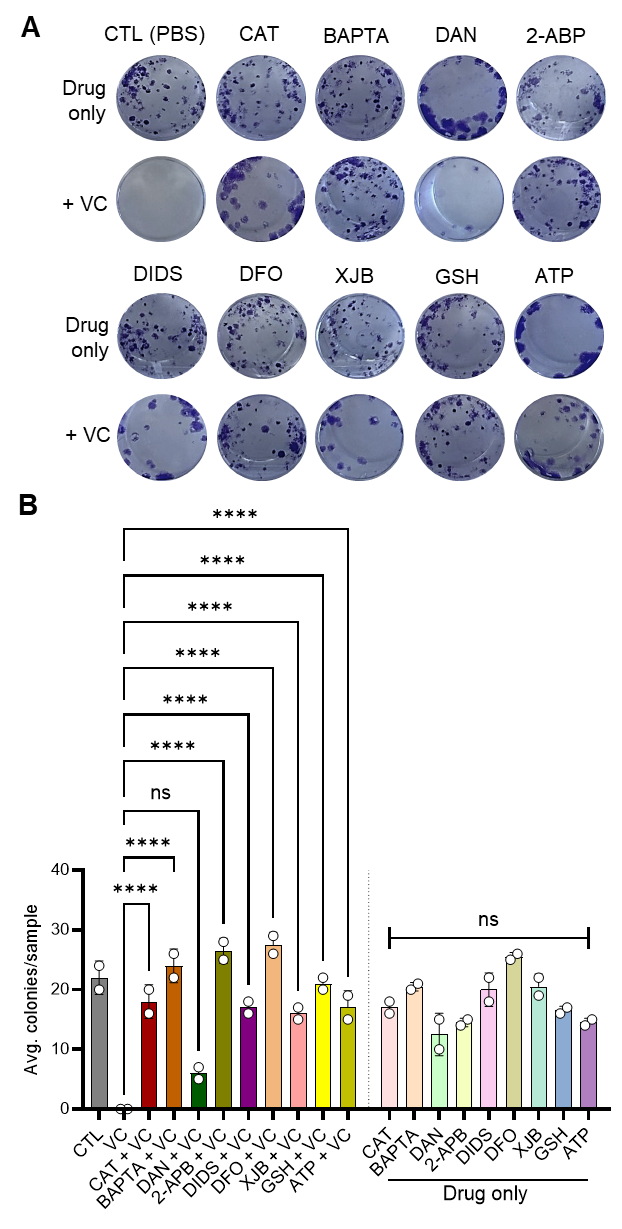


**Supplementary Fig. 9. Clonogenic cell survival analysis of the effect of various ion chelators and metabolic modulators on VC-induced cytotoxicity.** (**A**) U-2OS cells were treated with vitamin C (VC, 5 mM) for 3 hours and/or pre-treated with drugs (Catalase [CAT, 2000 U], BAPTA AM intracellular calcium inhibitor [BAPTA, 10 μM], Dantrolene [DAN, 60 μM], 2-Aminoethoxydiphenyl borate [2-APB, 100 μM], 4,4′-Diisothiocyano-2,2′-stilbenedisulfonic acid [DIDS, 100 μM], Deferoxamine [DFO, 100 μM], XJB-5-131 [XJB, 60 μM], glutathione [GSH, 100 μM], and ATP [100 μM]). Cells were trypsinized and seeded at a density of 200 cells/well for a clonogenic cell survival assay. After 10 days of incubation, cells were fixed with 4% PFA and stained with 0.5% crystal violet before imaging analysis. Representative images are shown from two independent experiments. (**B**) The number of colonies formed was analyzed using GraphPad software. ****p < 0.0001 by one-way ANOVA with Tukey's multiple comparisons test. ns., non-significant.

**Supplementary Fig. 10**


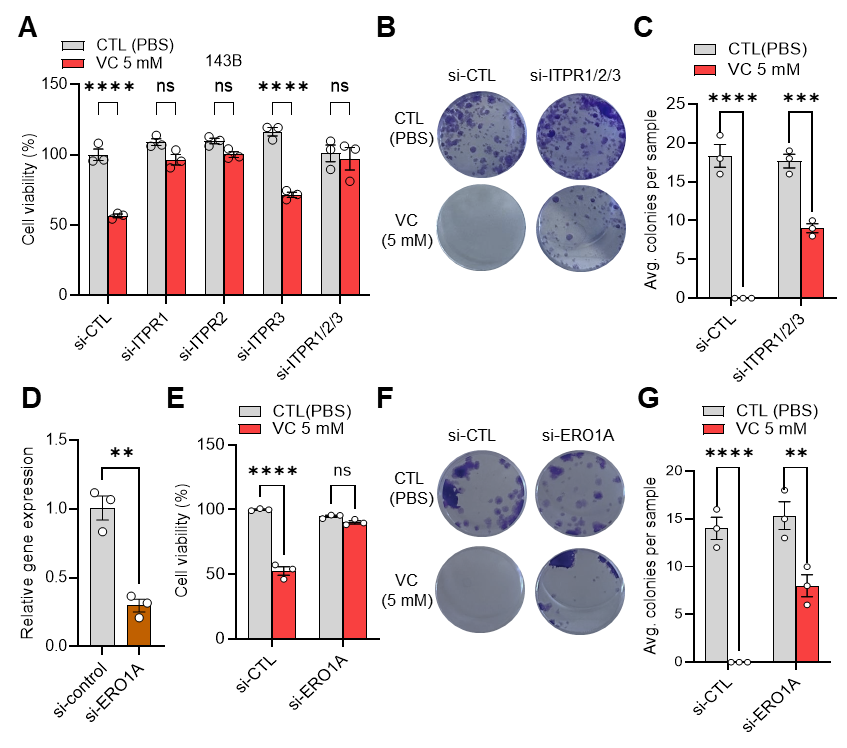


**Supplementary Fig. 10.** **The effects of ITPR or ERO1A gene knockdown on high-dose VC-induced cytotoxicity in human OS cells.** (**A**) Forty-eight hours after transfecting 143B cells with control siRNA (si-CTL) or siRNAs targeting individual ITPR isoforms (alone or in combination), the cells were exposed to 5 mM VC for 24 hours and subsequently subjected to cell viability assays. Statistical analysis using two-way ANOVA and Tukey’s multiple comparisons test revealed significant differences (****p < 0.001). ns., non-significant differences. (**B**) Clonogenic cell survival assay with ITPR RNAi. U-2OS cells were transfected with si-ITPR1/2/3. Forty-eight hours post-transfection, the cells were treated with 5 mM VC and/or PBS. After 3 hours, cells were trypsinized and seeded at a density of 200 cells/well for the clonogenic cell survival assay. After 10 days of incubation, cells were fixed with 4% PFA and stained with 0.5% crystal violet. (**C**) Number of colonies analyzed using Graphpad software. ***p < 0.001, ****p < 0.0001 by one-way ANOVA with Tukey's multiple comparisons test. (**D**) qPCR analysis to verify the efficiency of siRNA targeting ERO1A. Forty-eight hours post-transfection, RNA was harvested, and qPCR knockdown validation was performed. (**E**) The effects of *ERO1A* knocking-down on high-dose vitamin C-induced cytotoxicity in U-2OS cells. Forty-eight hours after transfection with control siRNA (si-CTL) or siRNA targeting *ERO1A*, cells were exposed to 5 mM VC for 24 hours and subsequently subjected to cell viability assays. (**F**) The si-*ERO1A* transfected cells were treated with VC and/or PBS. After 3 hours, cells were trypsinized and seeded at a density of 200 cells/well for the clonogenic cell survival assay. After 10 days of incubation, cells were fixed with 4% PFA and stained with 0.5% crystal violet. (G) Number of colonies analyzed using Graphpad software. **p < 0.001, ****p < 0.0001 by one-way ANOVA with Tukey's multiple comparisons test. ns., non-significant. Data are representative of two independent experiments and are shown as means ± SD (n = 3).

**Supplementary Fig. 11**


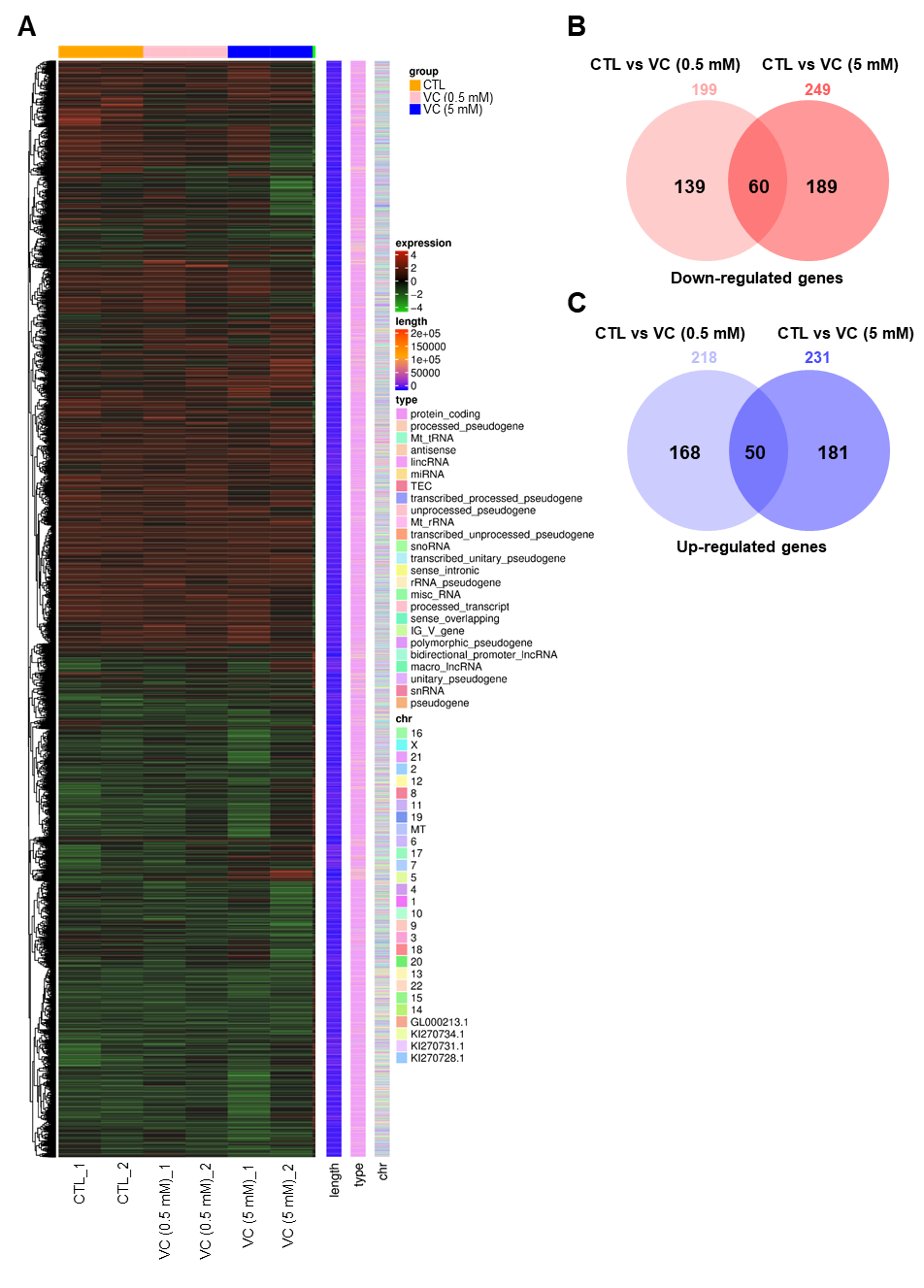


**Supplementary Fig. 11.** **Transcriptome analysis of U-2OS cells treated with and without VC.** (**A**) Heat map showing the differentially expressed genes in untreated U-2OS cells and cells treated with low (0.5 mM) or high (5 mM) doses of vitamin C (Supplementary data file 1). (**B, C**) Venn diagrams comparing the numbers of down-regulated (B) and up-regulated (C) genes in U-2OS cells treated with low (0.5 mM) or high (5 mM) doses of vitamin C (VC) relative to untreated control (CTL) cells.

**Supplementary Fig. 12**


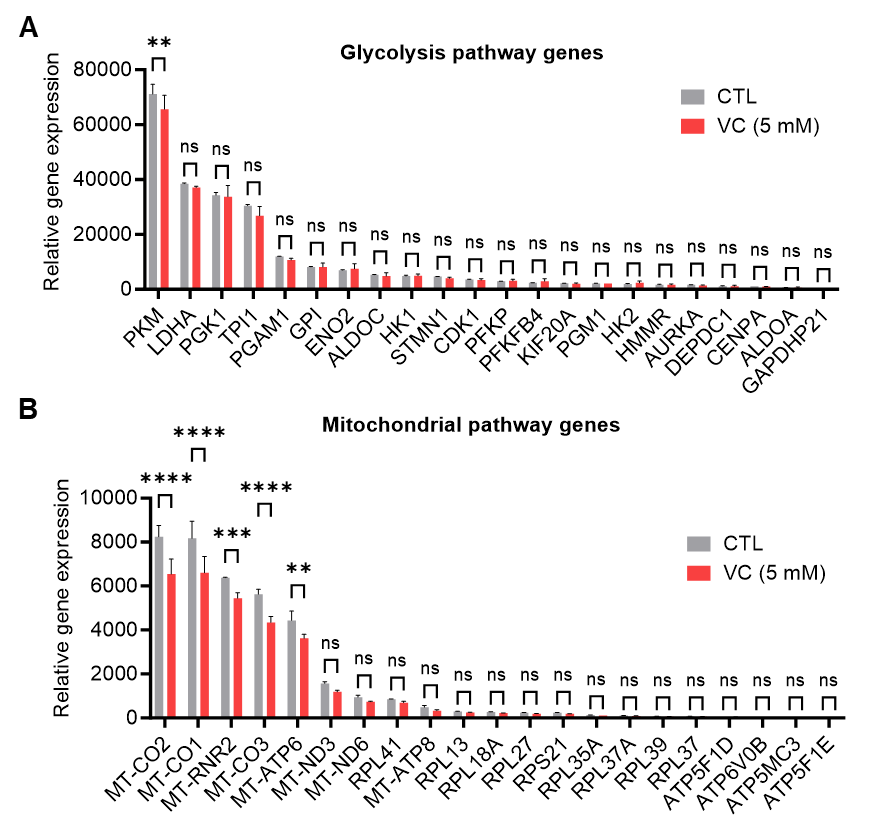


**Supplementary Fig. 12. Comparative analysis of the effects of high-dose vitamin C on the expressions of genes in the glycolysis and mitochondrial pathways in U-2OS cells.** (**A**) Comparative expression profile of representative glycolysis-related genes in control (CTL) and VC (5 mM)-treated U-2OS cells, analyzed from RNA Sequencing Data (Supplementary data file 1)**.** (**B**) Comparative expression profile of representative mitochondrial pathway genes in control (CTL) and VC (5 mM)-treated U-2OS cells**,** analyzed from RNA Sequencing Data (Supplementary data file 1). ***p* <0.005, ****p* <0.001, *****p* <0.0001 by two-way ANOVA with Tukey's multiple comparisons test; ns., non-significant.

**Supplementary Fig. 13**


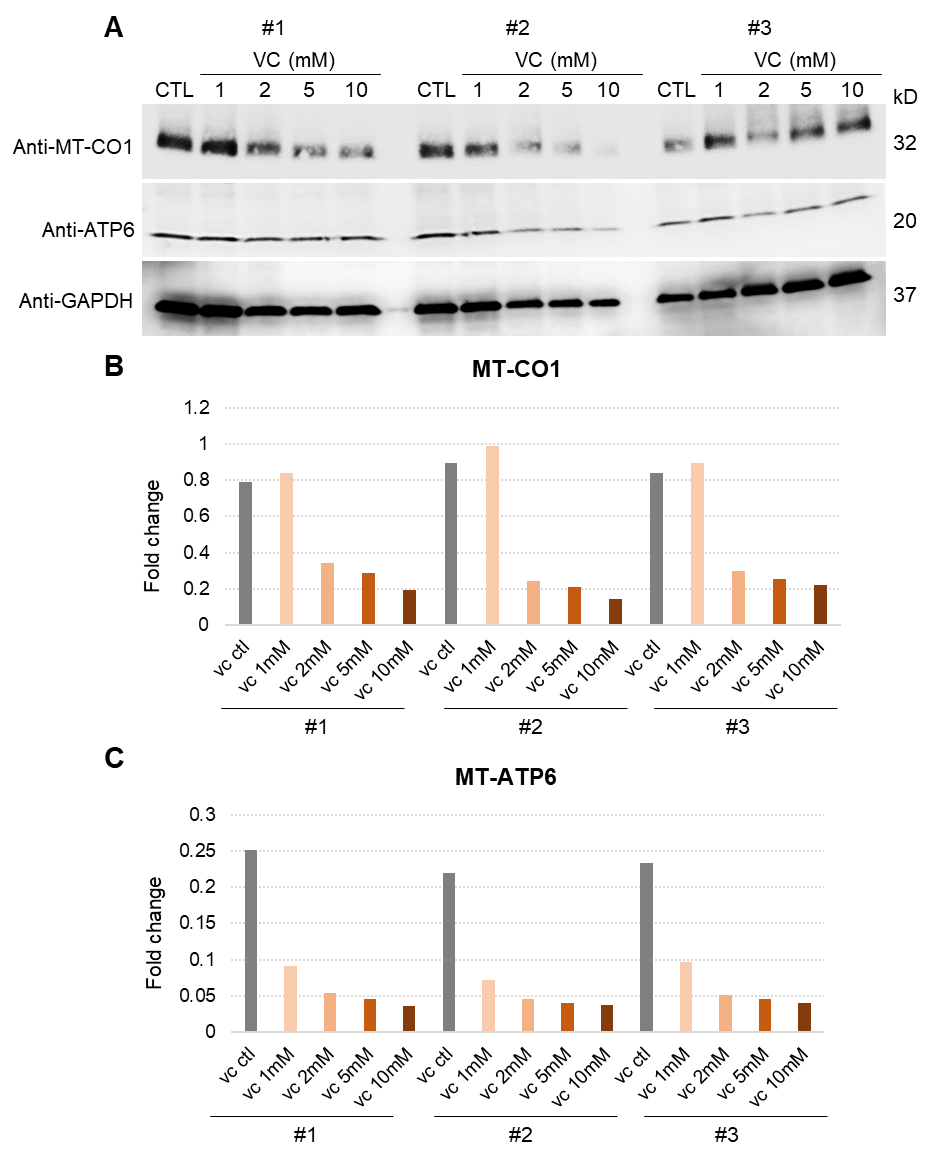


**Supplementary Fig. 13. Dose-dependent effects of vitamin C on mitochondrial gene expression.** (**A**) Western blot analysis illustrating the dose-dependent effects of VC (1 ~ 10 mM) on the protein expressions of MT-CO2 and MT-ATP6. (**B, C**) Statistical analysis of the Western blot data for the indicated proteins in A. Data were normalized to GAPDH and are presented as triplicate experiments.

**Supplementary Fig. 14**


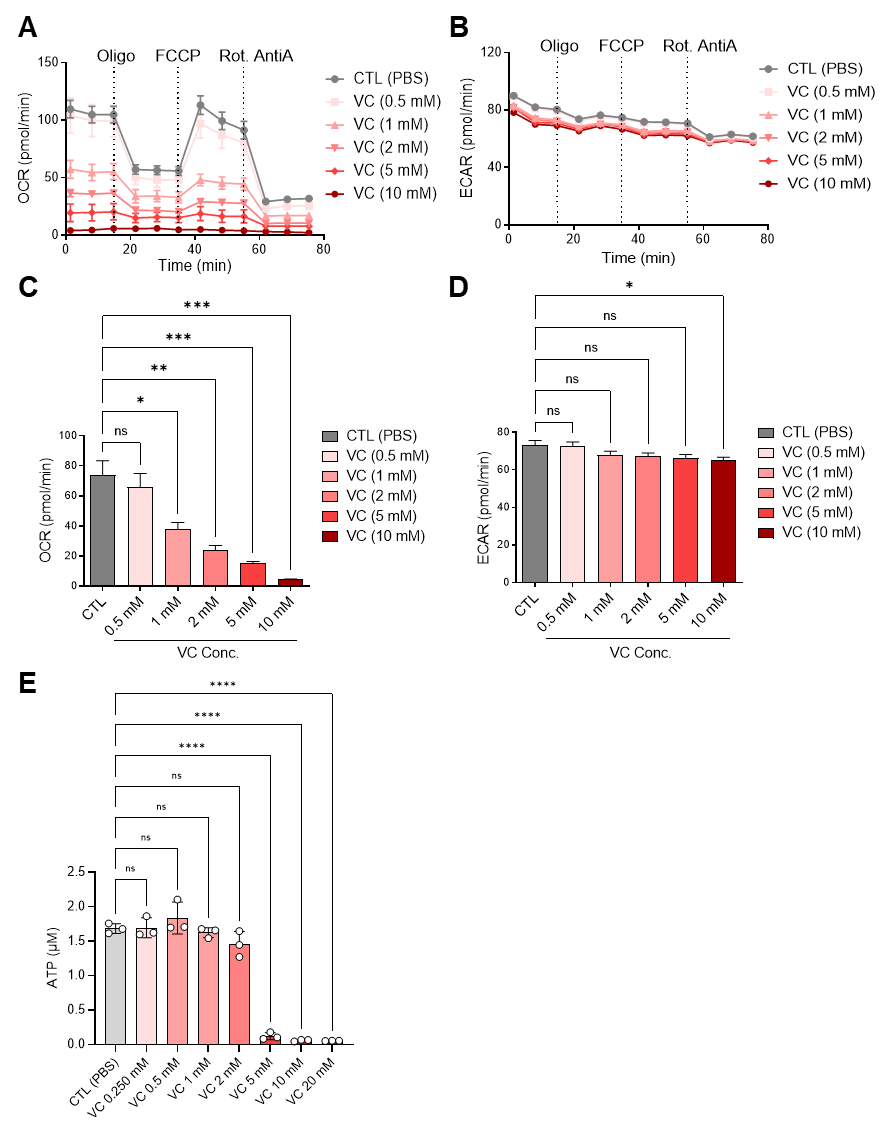


**Supplementary Fig. 14.** **Metabolic flux and ATP analyses of 143B cells treated with varying doses of vitamin C.** (**A-D**) Oxygen consumption rates (OCRs) (A, C) and extracellular acidification rates (ECARs) (B, D) were monitored to determine the dose-dependent effects of VC (0.5 ~ 10 mM) on mitochondrial respiration and glycolysis in 143B cells. For the OCR assays, all cells were exposed sequentially to oligomycin, FCCP, and rotenone plus antimycin A. **p* < 0.05, ****p* <0.001 by one-way ANOVA with Tukey's multiple comparisons test. Error bars indicate standard error of the mean (SEM), *n* = 6 per group. (**E**) ATP detection assays were performed in 143B cells treated with PBS or VC at the indicated concentrations (0.25 ~ 20 mM) for 6 hours.

**Supplementary Fig. 15**


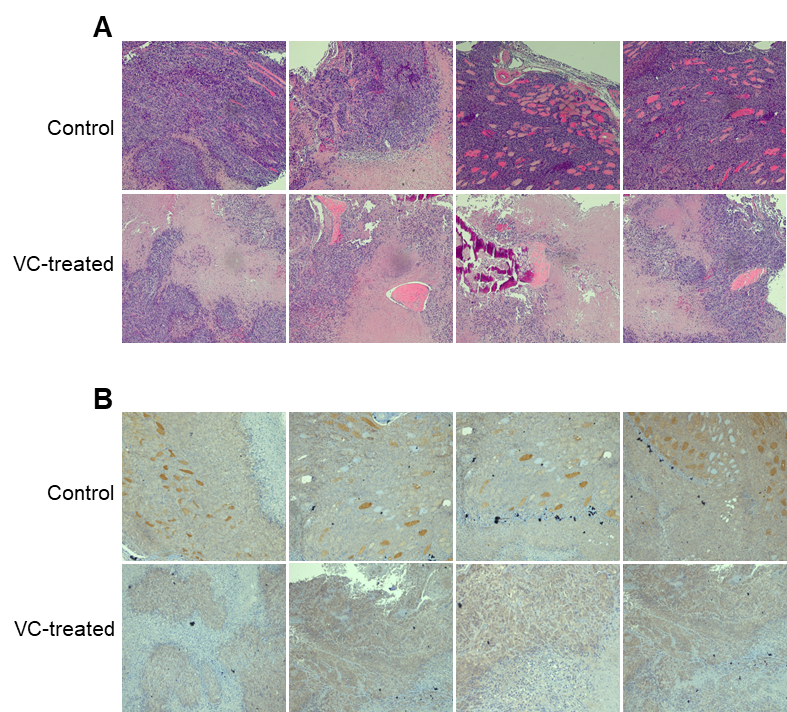


**Supplementary Fig. 15.** **Histological analysis of the effects of high-dose vitamin C on human OS xenograft tumors in mice.** (**A, B**) Representative images for hematoxylin and eosin staining (A) as well as immunohistochemical (IHC) analysis of MT-ATP6 expression using anti-MT-ATP6 antibody of tumor tissues harvested from vehicle (CTL) and VC (3.3g/kg)-treated mice.

**Supplementary Table 1.** qPCR primers for mitochondrial and ITPR genes.

| Target gene | Sequence |
| --- | --- |
| MT-CO1 | Forward sequence:  GACGTAGACACACGAGCATATTTCA  Reverse sequence:  AGGACATAGTGGAAGTGAGCTACAAC |
| MT-CO2 | Forward sequence:  ACAGATGCAATTCCCGGACGTCTA  Reverse sequence:  GGCATGAAACTGTGGTTTGCTCCA |
| MT-CO3 | Forward sequence:  ACTTCCACTCCATAACGCTCCTCA  Reverse sequence:  TGGCCTTGGTATGTGCTTTCTCGT |
| MT-ND3 | Forward sequence:  CCCTACCATGAGCCCTACAAACAA  Reverse sequence:  AGTCACTCATAGGCCAGACTTAGG |
| MT-ND6 | Forward sequence:  CAAACAATGTTCAACCAGTAACCACTAC  Reverse sequence:  ATATACTACAGCGATGGCTATTGAGGA |
| MT-ATP6 | Forward sequence:  TAGCCATACACAACACTAAAGGACGA  Reverse sequence:  GGGCATTTTTAATCTTAGAGCGAAA |
| ITPR1 | Forward sequence:  CCTGGTTGATGATCGTTGTGTT  Reverse sequence:  GCTTTTGGGCAGAGTAGCGGTT |
| ITPR2 | Forward sequence:  CACCTTGGGGTTAGTGGATGA  Reverse sequence:  CTCGGTGTGGTTCCCTTGT |
| ITPR3 | Forward sequence:  CCAAGCAGACTAAGCAGGACA  Reverse sequence:  ACACTGCCATACTTCACGACA |
| ERO1a | Forward sequence:  TGCTTCTGCCAGGTTAGTGG  Reverse sequence:  TCCACTGCTCCAAGTCGTTC |
